# An atlas of transcriptional, chromatin accessibility, and surface marker changes in human mesoderm development

**DOI:** 10.1038/sdata.2016.109

**Published:** 2016-12-20

**Authors:** Pang Wei Koh, Rahul Sinha, Amira A. Barkal, Rachel M. Morganti, Angela Chen, Irving L. Weissman, Lay Teng Ang, Anshul Kundaje, Kyle M. Loh

**Affiliations:** 1 Department of Genetics and Department of Computer Science, Stanford University, Stanford, California 94305, USA; 2 Department of Developmental Biology, Institute for Stem Cell Biology and Regenerative Medicine, Ludwig Center for Cancer Stem Cell Biology and Medicine, Stanford University School of Medicine, Stanford, California 94305, USA; 3 Stem Cell & Regenerative Biology Group, Genome Institute of Singapore, A*STAR, Singapore 138672, Singapore

**Keywords:** Pluripotency, Differentiation, High-throughput screening, Gene expression analysis

## Abstract

Mesoderm is the developmental precursor to myriad human tissues including bone, heart, and skeletal muscle. Unravelling the molecular events through which these lineages become diversified from one another is integral to developmental biology and understanding changes in cellular fate. To this end, we developed an *in vitro* system to differentiate human pluripotent stem cells through primitive streak intermediates into paraxial mesoderm and its derivatives (somites, sclerotome, dermomyotome) and separately, into lateral mesoderm and its derivatives (cardiac mesoderm). Whole-population and single-cell analyses of these purified populations of human mesoderm lineages through RNA-seq, ATAC-seq, and high-throughput surface marker screens illustrated how transcriptional changes co-occur with changes in open chromatin and surface marker landscapes throughout human mesoderm development. This molecular atlas will facilitate study of human mesoderm development (which cannot be interrogated *in vivo* due to restrictions on human embryo studies) and provides a broad resource for the study of gene regulation in development at the single-cell level, knowledge that might one day be exploited for regenerative medicine.

## Background & Summary

A longstanding goal of regenerative medicine has been to efficiently differentiate stem cells into pure, functional populations of desired cell types. This has been challenging to achieve in practice: many extant differentiation methods take weeks or months to complete and result in heterogeneous mixtures of the target lineage and other contaminating lineages. Difficulties in differentiating stem cells into desired cell-types *in vitro* might stem from incomplete knowledge of how stem cells naturally develop into these lineages during the course of embryonic development.

We focus here on human mesoderm development, which starts with the differentiation of pluripotent stem cells into the primitive streak (PS) and then into paraxial and lateral mesoderm^1–3^. Paraxial mesoderm subsequently buds off into tissue segments known as somites^4^, with dorsal somites (dermomyotome) giving rise to brown fat, skeletal muscle, and dorsal dermis, and ventral somites (sclerotome) yielding the bone and cartilage of the spine and ribs^5^. Separately, lateral mesoderm goes on to form limb bud mesoderm^6^ and cardiac mesoderm^7^, the latter of which generates cardiomyocytes and other heart constituents.

**Figure 1:**
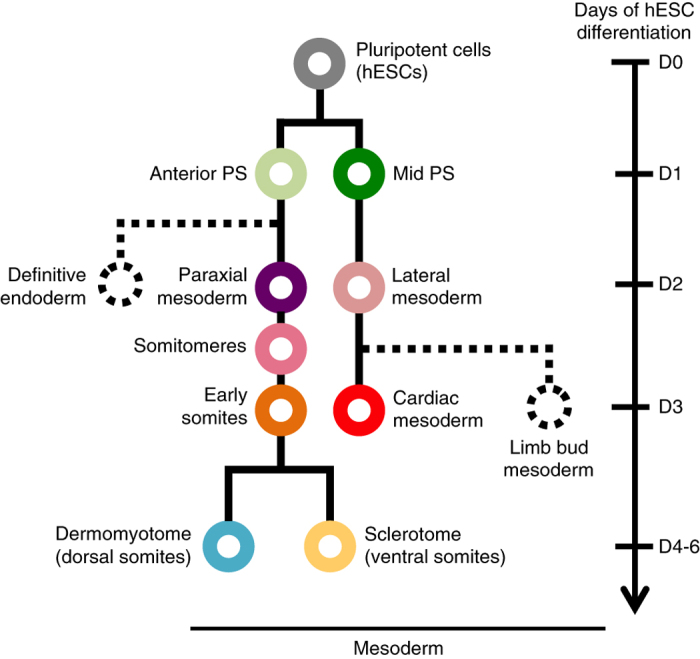
A schematic of human mesoderm development. We differentiate and profile each of the 10 cell types shown in color here, starting with pluripotent stem cells and ending in dermomyotome, sclerotome, and cardiac mesoderm.

Our related publication^8^ delineated a comprehensive roadmap for human mesoderm development that outlined key intermediate stages and defined the minimal combinations of extrinsic signals sufficient to induce differentiation at each stage. To elicit differentiation at defined stages, in addition to identifying the necessary inductive cues at each stage (as is typical), we also identified pathways leading to ‘unwanted’ cell fates and systematically repressed them at each lineage branchpoint. We used this strategy to efficiently differentiate pluripotent stem cells, through anterior and mid primitive streak, into paraxial and lateral mesoderm, and subsequently into somites, sclerotome, dermomytome, and cardiac mesoderm (Fig. 1). The identity and purity of these cell types was respectively assessed by transplantation into mouse models or single-cell gene expression profiling^8^.

Here we describe in detail the materials and methods used to generate and profile these distinct cell types, with an eye towards promoting reproducibility and reuse of our data. We focus on the biological methods used to generate the data; the computational pre- and post-processing of the data; and the technical validation of the quality of our data. In contrast, our related publication^8^ focused on experimentally validating the biological function and purity of the differentiated cell types and on extracting developmental insights from the data.

Our dataset comprises three main types of data -- gene expression, chromatin accessibility, and surface marker expression -- across 10 different cell types (pluripotent stem cells, anterior PS, mid PS, paraxial mesoderm, somitomeres, somites, sclerotome, dermomyotome, lateral mesoderm and cardiac mesoderm). For expression, we performed bulk-population RNA-seq as well as single-cell RNA-seq (using the Fluidigm C1 system) on a total of 651 cells spanning all lineages. Chromatin accessibility across the genome was measured by ATAC-seq^9^. For each lineage, two to six biological replicates were assayed for bulk-population RNA-seq and ATAC-seq. Finally, the expression of 332 cell-surface markers was ascertained on most lineages by means of high-throughput antibody screening.

Taken together, this dataset will constitute a useful resource for the study of human mesoderm development. For example, this dataset enabled us to identify novel marker genes in somitogenesis (a transient process which cannot be observed *in vivo* due to restrictions on the use of human embryos); identify the putative cell-of-origin for different subtypes of congenital scoliosis; and infer the activity of transcription factors at each stage of mesodermal development^8^. The data from the high-throughput surface marker screen will also be helpful in purifying desired cell types for transplantation or further study.

Moreover, we believe that this dataset will be useful as a broader resource for the analysis of a timecourse data, e.g., as a testing ground for algorithms that aim to reconstruct developmental paths from single-cell RNA-seq data^10,11^, or for the study of how changes in chromatin accessibility are correlated with, and are ultimately causative of, changes in gene expression across developmental time and space.

## Methods

We reproduce here the experimental protocols included in our related publication^8^, with added detail on our computational processing steps, RNA library construction, and surface marker screening. A list of all experiments reported here, together with accession codes of the corresponding data, can be found in Table 1 (available online only).

### Bulk-population RNA-seq

#### RNA extraction, library preparation and sequencing

For bulk-population RNA-seq, RNA was extracted from either whole cell populations or alternatively, cell subsets purified by fluorescence activated cell sorting (FACS). In brief, RNA was obtained from undifferentiated H7 hESCs (day 0 of *in vitro* differentiation), H7-derived anterior primitive streak populations (day 1), H7-derived mid primitive streak populations (day 1), H7-derived lateral mesoderm (day 2), H7-derived FACS-purified GARP+ cardiac mesoderm (day 3), H7-derived FACS-purified DLL1+ paraxial mesoderm populations (day 2), H7-derived day 3 early somite progenitor populations (day 3), H7-derived dermomyotome populations (day 5, treated with BMP4+CHIR99021+Vismodegib on days 4–5), and H7-derived FACS-purified PDGFR*α*+ sclerotome populations (day 6).

Total RNA from the above cell populations was isolated using Trizol (Thermo Fisher) as per the manufacturer's recommendations, with the additional use of linear polyacrylamide (Sigma) as a carrier to facilitate RNA precipitation. Purified total RNA was treated with 4 units of RQ1 RNase-free DNase (Promega) at 37 degrees Celsius for 1 h to remove trace amounts of genomic DNA. The DNase-treated total RNA was cleaned-up using the RNeasy Micro Kit (Qiagen). Subsequently, the integrity of extracted RNA was assayed by on-chip electrophoresis (Agilent Bioanalyzer) and only samples with a high RNA integrity (RIN) value were used for subsequent cDNA library preparation.

Purified total RNA (10–50 ng) was reverse-transcribed into cDNA and amplified using the Ovation RNA-seq System V2 (NuGEN). Amplified cDNA was sheared using the Covaris S2 (Covaris) with the following settings: total volume 120 *μ*l, duty cycle 10%, intensity 5, cycle/burst 100 and total time 2 min. The sheared cDNA was cleaned up using Agencourt Ampure XP beads (Beckman Coulter) to obtain cDNA fragments >=400 base pairs (bp). 500 ng of sheared and size-selected cDNA was used as input for library preparation using the NEBNext Ultra DNA Library Prep Kit for Illumina (New England BioLabs) as per the manufacturer's recommendations. Resulting libraries (fragment distribution: 300–700 bp; peak 500–550 bp) were pooled (multiplexed) and sequenced using either a HiSeq 4000 or NextSeq 500 (Illumina) at the Stanford Functional Genomics Facility to obtain 2×150 bp paired-end reads. For each RNA-seq library, the effectiveness of adapter ligation and the effective library concentration was determined by qPCR and Bioanalyzer (Agilent) prior to pooling and loading them onto the sequencers.

Each sample in our data constitutes a separate biological replicate. Bulk population RNA-seq libraries were prepared in three batches (Table 2).

#### Quantification and processing

Obtained RNA-seq reads were trimmed for base call quality (PHRED score >=21) and for adapter sequences (using Skewer^12^), and then were subsequently processed using a slightly-modified version of the ENCODE long RNA-seq pipeline for quantification of mRNA expression (https://www.encodeproject.org/rna-seq/long-rnas/)^13^. Specifically, reads were aligned to hg38 using STAR 2.4 (ref. 14); gene-level expression was then quantified using RSEM 1.2.21 (ref. 15). We only kept samples with at least 10,000,000 uniquely mapping reads and with at least 50% of reads uniquely mapping, which meant rejecting one sample (from sclerotome) out of 34. The numbers and percentages of uniquely mapping reads for each sample are listed in Table 2. The full parameter settings used can be found in our versions of STAR_RSEM.sh and STAR_RSEM_prep.py (see Code Availability below).

To facilitate global comparisons of gene expression levels across cell types, we first took the log2TPM (transcripts per million) values for each gene, before filtering out all genes where there was a difference of less than 2 (in log2TPM units, i.e., a 4-fold difference in expression) between the cell types with the highest and lowest expression. Next, we used ComBat with non-parametric priors^16^ (as implemented through the *sva* R package^17^) to correct for batch effects. This sometimes left small negative values for the expression of some genes, which we set to 0. The R Markdown script implementing this batch correction is bulkDataViz.Rmd.

For ease of use, we also prepared a spreadsheet with TPM values for each gene, augmented with the following information on each gene: 1) whether the gene product is present on the cell surface (GO code GO:0009986); 2) for each pair of adjacent conditions, whether the gene was differentially expressed between those conditions; and 3) the shrunken log-fold-change for that gene between those conditions. We provide (1) as a convenience to help in finding potential surface markers that were not included in our high-throughput screen (e.g., because an antibody was not available). (2) and (3) were calculated by DESeq2 (ref. 18) using batch information; genes were called as differentially expressed at a false discovery rate (FDR) of 0.1.

The raw data from the bulk-population RNA-seq can be found in [Data Citation 1]. A spreadsheet of TPM values can be found in [Data Citation 2]. The annotated spreadsheet, as described in the previous paragraph, is in [Data Citation 3].

### Single-cell RNA-seq

#### Library preparation and sequencing

Cells were briefly washed (DMEM/F12), dissociated (TrypLE Express), strained (100 μm filter), pelleted and re-suspended in DMEM/F12 for counting. Before single-cell capture, two quality control steps were implemented. First, cell size was estimated in order to determine whether cells should be loaded onto C1 capture arrays of either 10–17 μm or 17–25 μm size. Arrays were chosen for each lineage by estimating the median cell size of each given population on a flow cytometer on the basis of the FSC-W signal^19^ and choosing an array with an appropriate pore size to accommodate such cells. Second, to ensure the high viability of *in vitro*-differentiated cells prior to commencing single-cell RNA-seq, for each population a separate aliquot of cells was stained with 1.1 μM DAPI and analyzed by flow cytometry; for all cell populations that were used for single-cell RNA-seq, >98% of cells were viable (i.e., DAPI negative).

For single-cell capture, cells were diluted to a concentration of 1000 cells per μl, diluted in a 3:2 mixture of C1 Cell Suspension Reagent and DMEM/F12, and then loaded onto a Fluidigm C1 single-cell capture array chip for automated capture on a Fluidigm C1 Machine (Stanford Stem Cell Institute Genomics Core). 10–17 μm array chips were used for hESCs, day 1 anterior PS, day 2 sorted DLL1+ paraxial mesoderm, day 2.25 somitomeres, day 3 early somites, day 2 lateral mesoderm, day 3 sorted GARP+ cardiac mesoderm, day 5 central dermomyotome, and day 6 sorted PDGFRA+ sclerotome while a 17–25 μm array chip was used for day 1 mid PS.

After loading, the efficiency of single-cell capture was verified using an automated microscope that imaged each captured cell on the chip. Subsequent cell lysis, cDNA synthesis, and amplification was executed within each microfluidic chamber in the array chip in an automated fashion with the Fluidigm C1 machine using the reagents from SMARTer Ultra Low RNA Kit (Clontech, 634833), as per the manufacturers' instructions (Fluidigm, PN 100–7168 Rev. A2). The amplified cDNA from individual cells was harvested into a nuclease-free 96-well plate and diluted using the C1 harvesting reagent (Fluidigm). The concentration and integrity of amplified cDNA were assessed using a Fragment Analyzer (Advanced Analytical) in 96-well plate format. Amplified cDNAs from only those wells that (1) were not degraded and (2) originated from wells that were microscopically verified manually to contain a single cell, were carried forward for subsequent library construction. It is important to note that because of manual verification, we were able to effectively rule out doublets if captured in the medium (10–17 μm) or the large (17–25 μm) array chips.

A single-channel liquid handling robot, Mosquito X1(TTP Labtech), was used to simultaneously, 1) dilute amplified cDNAs from single cells from all lineages to a concentration range of 0.05–0.16 ng per μl with C1 Harvest Reagent (Fluidigm) as a diluent and 2) consolidate the diluted cDNA into 384 well plates. The diluted single-cell cDNAs were tagmented and converted to sequencing libraries in the 384 well plates using the Nextera XT DNA Sample Prep Kit (Illumina, FC-131–1096) in an automated fashion using another 16-channel pipetting robot, Mosquito HTS (TTP Labtech), and 384 distinct Illumina-compatible molecular barcodes. The resulting sequencing libraries from a single such 384 well plate were then pooled and cleaned up using Agencourt AMPure XP beads (Beckman Coulter). The pooled libraries were then analyzed for quality and concentration using Bioanalyzer (Agilent) and qPCR and loaded on a single lane of NextSeq 500 or two lanes of HiSeq 4000 to obtain 1–2 million 2×150 bp reads per cell. The reads obtained were trimmed for base call quality (PHRED score >=21) and the presence of adapter sequences using Skewer^12^.

#### Quantification and processing

We quantified single-cell gene expression using the ENCODE long RNA-seq pipeline (with the same parameter settings as employed for analysis of bulk-population RNA-seq). We only kept samples with at least 1 million uniquely mapped reads and at least 70% of reads uniquely mapping, which meant keeping data from 498 single cells out of 651. The numbers and percentages of uniquely mapping reads for each cell are listed in Table 3 (available online only).

We next filtered out genes with low or undetectable expression by only considering genes with least 20 cells (across all 498 retained cells) showing a log2 (TPM+1) value of at least 10 for that gene. As with the data from the bulk-population RNA-seq, when performing analyses comparing cell types to one another, we additionally filtered out genes whose log2 (TPM+1) values did not vary by a difference of at least 2 (i.e., a 4-fold difference in expression) between the cell types with the highest and lowest expressions.

The raw data from the single-cell RNA-seq can be found in [Data Citation 1]. A spreadsheet of TPM values can be found in [Data Citation 2].

### ATAC-seq

#### Library preparation and sequencing

ATAC-seq was performed as described previously^9^, with minor modifications. In brief, for each replicate, 50,000 cells were lysed in lysis buffer containing 0.01% IGEPAL CA-630 (Sigma, I8896) to obtain nuclei, which were directly used in the Tn5 transposition reaction (reagents from Nextera DNA Sample Preparation Kit; Illumina, FC-121–1030). Immediately following transposition, DNA fragments were purified (MinElute Kit, Qiagen) and PCR amplified for a total of 12–13 cycles using previously-designed primers that included Illumina compatible adapters and barcodes^9^. The resulting ATAC-seq libraries were purified (MinElute Kit, Qiagen) and pooled, and final library-pool concentrations were assessed (Bioanalyzer) prior to next-generation sequencing. The quality of ATAC-seq libraries was confirmed by a shallow sequencing run using a MiSeq v3 (Stanford Functional Genomics Facility, 2×75 bp reads) before deep sequencing was performed on a NextSeq 500 (2×75 bp reads). Two replicates were analyzed per cell-type.

#### Quantification and processing

We used the ATAqC pipeline^20^ to process the ATAC-seq reads, starting with adapter trimming and then alignment to hg19 (Bowtie2 (ref. 21)). While we used hg38 for RNA-seq alignment, we opted for hg19 for ATAC-seq because of the availability of a curated blacklist of artifactual regions in hg19 (ref. 13). We then filtered out reads based on a variety of criteria (excluding unmapped reads, mate-unmapped reads, secondary alignments, duplicates (using Picard's MarkDuplicates^22^), multi-mapping reads (MAPQ<30), and mitochondrial reads), retaining only high-read-quality, properly-paired reads.

Two biological replicates were assayed by ATAC-seq for each cell-type. As the post-filtering sequencing depth varied between replicates and cell types, we subsampled each replicate to a maximum of 35 M uniquely-mapping reads (post-filtering) to improve comparability between samples. We next used MACS2 (ref. 23) to call peaks for each replicate, with a relaxed false discovery rate (FDR) threshold of 0.01, and then created a unified peak list for each cell type by selecting only peaks that were reproducible between both replicates. This was done through an irreproducible discovery rate (IDR) analysis^24^, similar to what was previously described by the ENCODE Consortium^25^. In brief, the IDR method takes in peak calls from a pair of replicates, filters out all peaks that only appear in one replicate, and then uses a copula mixture model to model the remaining peaks as belonging to either a reproducible ‘signal’ population or an irreproducible ‘noise’ population’. We used an IDR threshold of 0.1, i.e., we only retained peaks that were deemed to have come from the ‘signal’ population with a probability of more than 0.9 after a multiple testing correction. Finally, we filtered out all peaks that appeared in the aforementioned blacklist of artifactual regions in hg19 (https://www.encodeproject.org/annotations/ENCSR636HFF/).

We note that this ATAC-seq analysis pipeline is an improved version of the one used for analysis in our related publication^8^. In particular, here we adjusted the IDR threshold, the shift size parameter for MACS2, and a multi-mapping parameter, resulting in increased sensitivity for peak detection.

To obtain a universal list of peaks across all cell-types, we used BEDtools^26^ to merge the lists of filtered, reproducible peaks for each cell-type, resulting in a total of 166,256 peaks. For each cell-type, we then pooled its two biological replicates together and called peaks (MACS2) on the pooled reads. To obtain a single measure of confidence at each peak P in the universal list for each cell-type C, we took the highest −log10 *P*-value out of all peaks in the pooled replicates for C that intersected with P.

The raw ATAC-seq data can be found in [Data Citation 1]. The peak calls can be found in [Data Citation 2]. ATAC-seq metadata is tabulated in Table 4 (available online only).

### High-throughput surface marker screening

High-throughput, antibody-based screening of surface markers expressed on various mesodermal progenitors was performed as described in our related publication^8^ and explained in further detail here. The following lineages, derived from the indicated embryonic stem cell lines, were screened using this approach: undifferentiated H7 hESCs (‘undifferentiated hESCs’), H7-derived day 2 paraxial mesoderm (‘paraxial mesoderm’), H7-derived day 3 early somite progenitors (‘early somite’), H7-derived day 5 dermomyotome (‘dermomyotome’), H7-derived day 6 sclerotome (‘sclerotome’), *MIXL1-GFP* reporter HES3 hESC-derived day 1 anterior primitive streak (‘primitive streak’) and finally, *NKX2.5-GFP* reporter HES3 hESC-derived day 3 cardiac mesoderm (‘cardiac mesoderm’). 10–70 million cells of each lineage were used in each surface-marker screen. Due to limited resources, we did not include mid primitive streak and lateral mesoderm in this screen.

Prior to antibody staining, hESCs or their differentiated mesodermal progeny were dissociated by brief 37 C incubation in TrypLE Express (Gibco). TrypLE Express was chosen as a dissociation reagent, as it has been previously shown to minimally cleave cell-surface epitopes^27^, which would otherwise confound surface marker screening data. After cell detachment, they were washed off plates in a large excess of DMEM/F12 to neutralize the dissociation reagent, filtered to remove large cell clumps, pelleted by centrifugation, and re-suspended in approximately 30 ml of Cell Suspension Buffer (Biolegend).

To conduct antibody screening, a multichannel pipette was used to plate the cell suspension into individual wells of four 96-well plates, each well containing a distinct PE-conjugated antibody against a human cell-surface antigen, altogether totaling 332 unique cell-surface markers across multiple 96-well plates (LEGENDScreen PE-Conjugated Human Antibody Plates; Biolegend, 700001). Cells were stained with respective antibodies for 30 min at 4 C, washed twice with Cell Staining Buffer and then finally re-suspended in Cell Staining Buffer containing 1.1 μM DAPI (Biolegend) as a viability dye before analysis on an LSR Fortessa (Stanford Stem Cell Institute FACS Core). Stained cells were not fixed prior to FACS analysis.

The percentage of viable (DAPI-negative cells) for each lineage that expressed each given surface marker was determined by rigorously gating the PE fluorescent signal such that no more than several percent of negative control cells (unstained cells or cells that were stained with an isotype control antibody directed against no known cellular antigen) were regarded positive. For analysis of surface-marker expression on *MIXL1-GFP* reporter HES3 hESC-derived primitive streak or *NKX2.5-GFP* reporter HES3 hESC-derived day 3 cardiac mesoderm, cells were respectively pre-gated on the MIXL1-GFP+ and NKX2.5-GFP+ fractions before analysis of PE signal intensity. Multicolor compensation was conducted to control for fluorescent bleedthrough between the PE and GFP channels.

A table with the percentage of viable cells in each lineage that expressed each given surface marker can be found in [Data Citation 4]. Metadata for the surface marker screen is tabulated in Table 5 (available online only).

### Code availability

All custom code used in this work is available at https://github.com/kundajelab/mesoderm. This includes R Markdown files that reproduce the figures in this paper.

For RNA-seq processing and quantification, we used STAR 2.4 (ref. 14), RSEM 1.2.21 (ref. 15), and Skewer 0.1.127 (ref. 12). The full parameter settings for STAR and RSEM can be found in STAR_RSEM.sh and STAR_RSEM_prep.py in the Github repository above. For bulk-population RNA-seq read processing, we used the following parameters for Skewer:

-x
AGATCGGAAGAGCACACGTCTGAACTCCAGTCACNNNNNNATCTCGTATGCCGTCTTCTGCTTG

-y
AGATCGGAAGAGCGTCGTGTAGGGAAAGAGTGTAGATCTCGGTGGTCGCCGTATCATT

-t 16 -q 21 -l 21 -n -u -f sanger

For single-cell RNA-seq read processing, we used the following parameters for Skewer:

-x
CTGTCTCTTATACACATCTCCGAGCCCACGAGACNNNNNNNNATCTCGTATGCCGTCTTCTGCTTG

-y
CTGTCTCTTATACACATCTGACGCTGCCGACGANNNNNNNNGTGTAGATCTCGGTGGTCGCCGTATCATT

-t 16 -q 21 -l 21 -n -u -f sanger

For ATAC-seq processing, we used commit 9077b9... of the ATAqC pipeline^20^. In turn, this used MACS2 2.1.0 (ref. 23) and Bowtie2 2.2.6 (ref. 21).

### Differentiation

#### Human pluripotent stem cell culture

H7, *MIXL1-GFP* HES3, *NKX2.5-GFP* HES3, *SOX17-mCherry* H9, *pCAG-GFP* H7, *EF1A-BCL2-2A-GFP* H9 and *UBC-Luciferase-2A-tdTomato*;* EF1A-BCL2-2A-GFP* H9 hESCs and BJC1 hiPSCs were routinely propagated feeder-free in mTeSR1 medium (StemCell Technologies)+1% penicillin/streptomycin (Gibco) on cell culture plastics coated with Geltrex basement membrane matrix (Gibco). Undifferentiated human pluripotent stem cells (hPSCs) were maintained at high quality with particular care to avoid any spontaneous differentiation, which would confound downstream differentiation. Unless otherwise indicated, the majority of experiments performed in this study were conducted using H7 hESCs, including all bulk-population RNA-seq, single-cell RNA-seq, and ATAC-seq experiments.

#### Directed differentiation in defined medium

Partially-confluent wells of undifferentiated hPSCs were dissociated into very fine clumps using Accutase (Gibco) and sparsely passaged 1:12-1:20 onto new Geltrex-coated cell culture plates in mTeSR1 supplemented with 1 μM thiazovivin (Tocris; a ROCK inhibitor to prevent cell death after dissociation) overnight. Seeding hPSCs sparsely prior to differentiation was critical to prevent cellular overgrowth during differentiation, especially during long-duration differentiation. hPSCs were allowed to plate overnight. The following morning, they were briefly washed (in DMEM/F12) before the addition of differentiation medium. All differentiation was conducted in serum-free, feeder-free and monolayer conditions in chemically-defined CDM2 basal medium.

The composition of CDM2 basal medium^28^ was as follows: 50% IMDM (+GlutaMAX, +HEPES, +Sodium Bicarbonate; Gibco, 31980-097)+50% F12 (+GlutaMAX; Gibco, 31765-092)+1 mgml^−1^ polyvinyl alcohol (Sigma, P8136-250G)+1% v/v concentrated lipids (Gibco, 11905-031)+450 μM monothioglycerol (Sigma, M6145)+0.7 μgml^−1^ insulin (Roche, 1376497)+15 μgml^−1^ transferrin (Roche, 652202)+1% v/v penicillin/streptomycin (Gibco). Polyvinyl alcohol was brought into solution by gentle warming and magnetic stirring in IMDM/F12 media before addition of additional culture supplements.

#### Primitive streak induction

As previously described^8^, after overnight plating, hPSCs were briefly washed (with DMEM/F12) and then differentiated into either anterior primitive streak (30 ngml^−1^ Activin A+4 μM CHIR99021+20 ngml^−1^ FGF2+100 nM PIK90; for subsequent paraxial mesoderm induction) or mid primitive streak (30 mgml^−1^ Activin A+40 ngml^−1^ BMP4+6 μM CHIR99021+20 ngml^−1^ FGF2+100 nM PIK90; for subsequent cardiac mesoderm induction) for 24 h. Though both types of primitive streak broadly expressed pan-primitive streak markers (e.g., *MIXL1* and *BRACHYURY*), anterior and mid primitive streak lineages were distinguished by expression of distinct region-specific markers and differing developmental competence to develop into downstream lineages^8^.

Subsequently, day 1 anterior primitive streak was briefly washed (DMEM/F12) and differentiated towards day 2 paraxial mesoderm for 24 h (1 μM A-83-01+3 μM CHIR99021+250 nM LDN-193189 [DM3189]+20 ngml^−1^ FGF2). Separately, day 1 mid primitive streak was differentiated towards day 2 lateral mesoderm for 24 h (1 μM A-83-01+30 ngml^−1^ BMP4+1 μM C59; with 2 μM SB-505124 sometimes used instead of A-83-01)^8^.

#### Paraxial mesoderm downstream differentiation

Day 2 paraxial mesoderm was briefly washed (DMEM/F12) and further differentiated into day 3 early somite precursors for 24 hs (1 μM A-83-01+250 nM LDN-193189+1 μM C59+500 nM PD0325901). Subsequently, day 3 early somites were dorsoventrally patterned into either ventral somites/sclerotome (5 nM 21 K+1 μM C59) or dorsal somites/dermomyotome (3 μM CHIR99021+150 nM Vismodegib). Sclerotome induction was conducted for 48–72 h (leading to day 5–6 ventral somite progenitors). For dermomyotome induction, sometimes dermomyotome was induced in the presence of 50 ngml^−1^ BMP4 to upregulate *PAX7* after 48 h of BMP4+CHIR99021+Vismodegib differentiation (leading to day 5 dermomyotome progenitors)^8^. Media was changed every 24 h for all steps. The small-molecule Hedgehog agonist 21 K^29^ was commercially synthesized.

#### Lateral/cardiac downstream differentiation

Day 2 lateral mesoderm was differentiated into day 4 cardiac mesoderm by treating them with 1 μM A8301+30 ngml^−1^ BMP4+1 μM C59+20 ngml^−1^ FGF2 for 48 h, or alternatively, with 1 μM A8301+30 ngml^−1^ BMP4+20 ngml^−1^ FGF2 for 24 h followed by 25 ngml^−1^ Activin+30 ngml^−1^ BMP4+1 μM C59 for the next 24 h. Subsequently, day 4 cardiac mesoderm was briefly washed (DMEM/F12) and treated with 30 ngml^−1^ BMP4+1 μM XAV939+200 μg/ml 2-phospho-ascorbic acid (Sigma) for 48–96 h to yield day 6–8 cardiomyocyte-containing populations. Spontaneously contracting cardiomyocyte foci were evident from day 8 onwards^8^.

## Data Records

The raw RNA-seq data (bulk-population and single-cell) and ATAC-seq data can be found at SRA under BioProject PRJNA319573 (accession number SRP073808) [Data Citation 1].

Reproducible peak calls on our ATAC-seq data, as well as transcript per million (TPM) values for each gene and sample in our bulk-population and single-cell RNA-seq data, can be found at GEO under accession number GSE85066 [Data Citation 2]. Bulk-population RNA-seq metadata and mapping statistics can be found in Table 2, while single-cell RNA-seq mapping statistics are in Table 3 (available online only). ATAC-seq metadata can be found in Table 4 (available online only).

For ease of usage, the collated bulk-population RNA-seq data can be viewed at http://cs.stanford.edu/∼zhenghao/mesoderm_gene_atlas. As described above, an augmented spreadsheet with TPM values for each gene (for bulk-population RNA-seq data) and additional annotations about whether each gene corresponds to a potential cell surface marker and whether the gene was differentially expressed between conditions can be found on Figshare with DOI 10.6084/m9.figshare.3842835 [Data Citation 3].

Processed surface marker data (a table with the percentage of cells expressing each marker in each cell type) can be found on Figshare with DOI 10.6084/m9.figshare.3505817 [Data Citation 4]. Surface marker screening metadata is in Table 5 (available online only).

The full set of ATAC-seq quality control graphs for all of our samples can be found on Figshare with DOI 10.6084/m9.figshare.3507167 [Data Citation 5].

## Technical Validation

### Bulk-population RNA-seq

As mentioned above (see Methods), we only analyzed samples with at least 10,000,000 uniquely mapping reads and with at least 50% of reads uniquely mapping. On average, each sample had 45 M uniquely mapping reads with 69% of reads uniquely mapping; full numbers and percentages are in Table 2.

**Figure 2:**
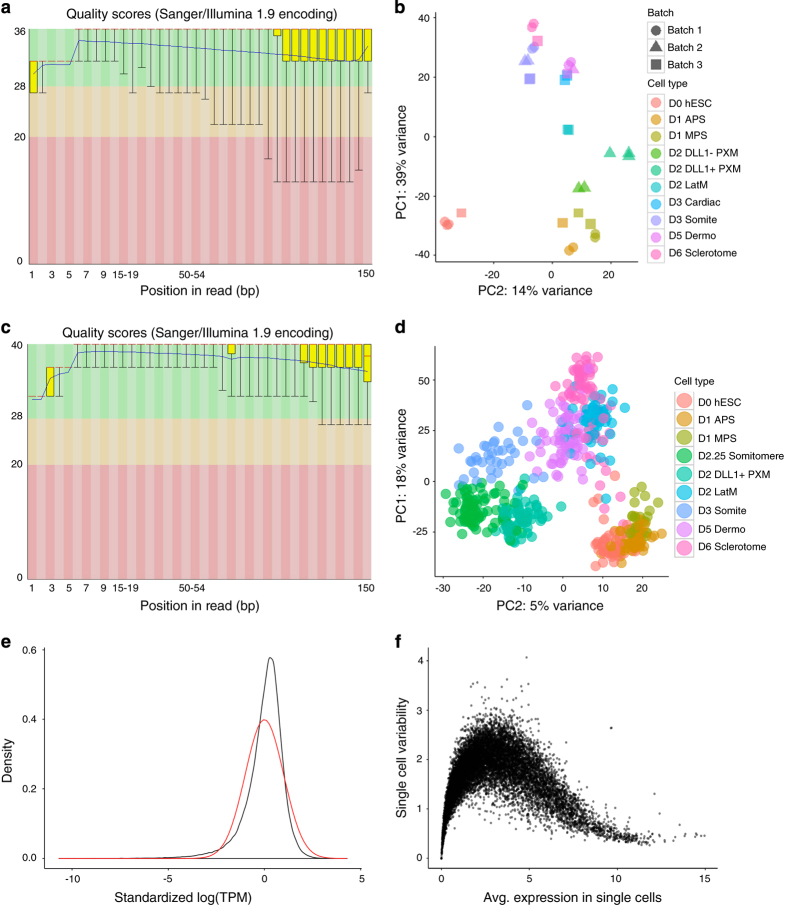
RNA-seq data quality and visualization. (**a**). Bulk-population RNA-seq FastQC quality scores across read position, shown for a representative sample (D0 hESC). (**b**). PCA plot of bulk-population RNA-seq data, based on the top 500 genes by variance across all samples, and using log_2_ TPM values. (**c**). Single-cell RNA-seq FastQC quality scores across read position, shown for a representative sample (D2.25 somitomeres). (**d**). PCA plot of single-cell RNA-seq data, based on the top 500 genes by variance across all cells, and using log_2_ TPM values. (**e**). Black: Plot of density against standardized log_2_ TPM for single-cell RNA-seq data across all genes in all cells, after removing zeroes. Red: Fitted normal distribution. (**f**). Plot of single-cell variability (s.d.) against mean expression value for each gene, shown for a representative cell type (paraxial mesoderm).

We used FastQC^30^ to measure the per-base sequence quality for each of our bulk-population RNA-seq experiments. All of the samples passed this quality check (i.e., for each base, the distribution of quality scores had a lower quartile of more than 10 and a median of more than 25). We show a representative FastQC plot (of the first sample we assayed) in Fig. 2a.

We also used principal component analysis (PCA) to visually inspect how the samples were distributed in log_2_(TPM) space. Applying PCA to the 500 genes with highest variance across all samples revealed the presence of batch effects. After correcting for batch effects (see Methods), the PCA plot showed tight clustering (Fig. 2b) among samples and implicitly suggested the developmental trajectory of the cells, starting from human embryonic stem cells in the bottom left and moving upwards towards cardiac mesoderm and somites and their derivatives. An R Markdown script to reproduce Fig. 2b is provided in bulkDataViz.Rmd in our Github repository.

Lastly, in our related publication^8^, we independently validated our RNA-seq results by qPCR. Specifically, we conducted qPCR to measure the mRNA expression levels of key genes known to be lineage markers for the various cell types in our study (e.g., *TBX6* and *MSGN1* for paraxial mesoderm; *PARAXIS*, *MEOX1*, and *FOXC2* in the somites). These qPCR expression patterns corroborated our RNA-seq results^8^.

### Single-cell RNA-seq

Before sequencing, we used an automated microscope to image each of the cell-capture wells on our Fluidigm C1 chips and manually inspected each image; for subsequent single-cell RNA-seq library construction we only used libraries from wells that contained exactly one cell. After sequencing, we filtered out cells with fewer than 1 million uniquely mapping reads or with fewer than 70% of reads uniquely mapping. Unfortunately, under these stringent selection critera, all cardiac mesoderm cell RNA-seq libraries were discarded; we ultimately retained 498 single cells out of 651. Full statistics of the cells are provided in Table 3 (available online only).

As with the bulk-population RNA-seq data, we used FastQC^30^ to check the per-base sequence quality of each experiment. All of the cells passed this quality check (i.e., for each base, the distribution of quality scores had a lower quartile of more than 10 and a median of more than 25), with a representative FastQC plot in Fig. 2c.

To visualize the distribution of single cells, we once again used PCA on the 500 genes with highest variance (Fig. 2d) in log_2_(TPM) space. As expected, the single-cell RNA-seq libraries separated by cell type, with cell types that are closer to each other biologically (and temporally) tending to cluster together. We note that each cell type was loaded onto a different Fluidigm C1 chip, and due to resource constraints we were only able to use one chip per cell type. This means that cell type is perfectly confounded with chip in our single-cell RNA-seq experiments, and in particular, we cannot tell from the PCA the degree to which batch/chip effects are responsible for the observed separation between cell types.

To tackle this problem, for each cell type, we measured the overall Pearson correlation between the average expression in the single cells and the corresponding average expression in the bulk-population RNA-seq experiments, all in log2 TPM units. On average, correlation was 0.82, varying from 0.76 to 0.87 depending on cell type. To ensure that this behavior was not driven solely by housekeeping genes, we looked at key marker genes expressed across our cell types (e.g., *MIXL1* and *BRACHYURY* in primitive streak; *MSGN1* and *DLL3* in paraxial mesoderm; *HAND1* and *FOXF1* in lateral mesoderm; *HOPX* in somitomeres; *FOXC2* and *PAX9* in sclerotome). Single-cell RNA-seq expression patterns of these archetypic marker genes were consistent with independent measures from bulk-population RNA-seq, qPCR, flow cytometry, and immunostaining (data in (ref. 8)).

As technical checks, we also examined the distribution of TPM values across all genes and cells. This followed a roughly log-normal distribution (Fig. 2e) after removing zeros, as expected. Finally, for each cell type, we plotted the standard deviation of each gene against its mean expression value (shown for paraxial mesoderm in Fig. 2f), obtaining for each cell type an expected curve where standard deviation is lowest when average expression is very low (because the expression of the gene in each cell is close to zero) or very high (because high expression translates into a large number of reads, allowing us to reduce technical variation from sampling error).

The script to reproduce Fig. 2d–f is provided scDataViz.Rmd. The correlation between average expression in single cells and the bulk population can be analyzed by running scAverageCorrelation.r.

### ATAC-seq

Through the ATAqC pipeline^20^, we calculated a variety of quality metrics to validate our ATAC-seq data. First, we looked at how many reads remained in each replicate after removing reads that did not successfully align, multi-mapping reads, duplicate reads, and mitochondrial reads. We had two replicates per cell type, and on average, each replicate had 46 M reads remaining, enough to robustly call peaks.

**Figure 3:**
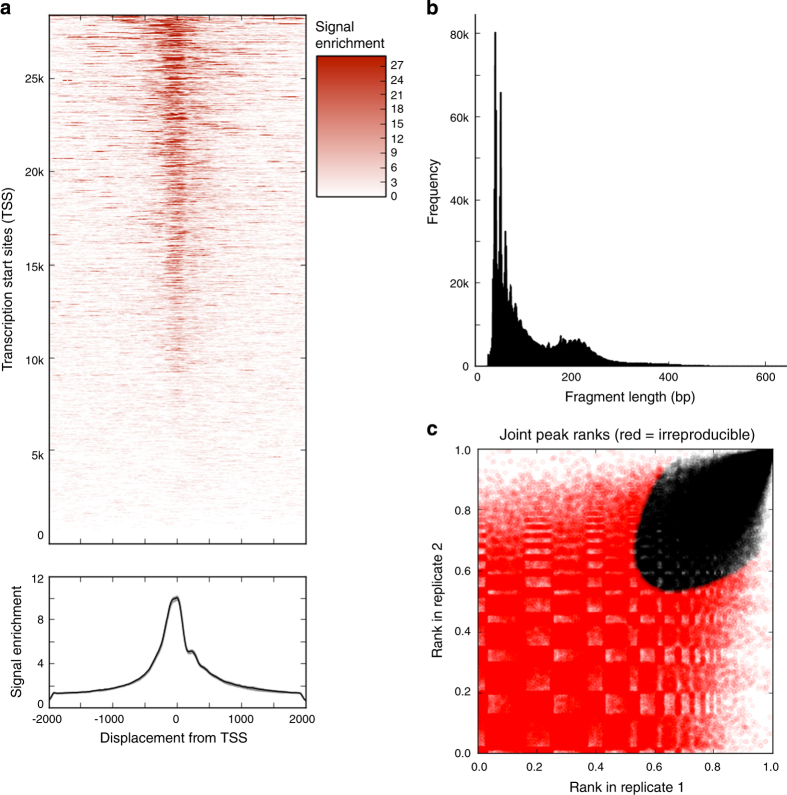
ATAC-seq data quality metrics. (**a**). Enrichment of ATAC-seq signal around transcription start sites (TSS), shown for a representative sample (lateral mesoderm). Top: enrichment around individual TSS. Bottom: aggregated enrichment around all TSS's. (**b**). Fragment length distribution of ATAC-seq reads from a representative sample (lateral mesoderm). Most of the reads fall into the nucleosome-free region (<150 bp) and a clear mono-nucleosome peak can be seen. (**c**). Irreproducible rate (IDR) analysis of ATAC-seq peaks from lateral mesoderm. The scatter plot shows one point for every peak, with its location representing in rank in each replicate. For downstream analysis, we only consider peaks shown in black (reproducible at an IDR rate of 0.1), which have ranks that are consistent between replicates.

We then looked at the fragment length distribution of the remaining reads; we show a representative plot from lateral mesoderm in Fig. 3a. A ‘good’ ATAC-seq experiment will have a majority of reads falling in the nucleosome-free region (NFR), with a mono-nucleosomal peak representing reads that cut on both sides of a nucleosome (≈200 bp in length). All of our samples displayed a mono-nucleosome peak, with 60–70% of reads falling in the NFR.

We also studied the enrichment of reads falling into transcription start sites (TSS), as TSS are known to be open chromatin sites (Fig. 3b; lateral mesoderm). On average, the enrichment of reads at TSS was 10.4x, with a range from 4.6x to 22.3x.

Next, we looked at the number of peaks called across each replicate. Because of the variability in quality across the experiments (e.g., some experiments had a higher TSS enrichment and/or more reads), we first subsampled each replicate to have a maximum of 35 M reads (post-filtering). We then used MACS2 (ref. 23) to call peaks on each replicate independently, before using an IDR analysis^24^ to identify peaks that were reproducible between the two replicates for each cell type (Fig. 3c). Using an IDR threshold of 0.1, we found an average of 91 K reproducible peaks per cell type.

Full statistics and metadata for each replicate is provided in Table 4 (available online only), including additional quality metrics such as library complexity metrics, the fraction of NFR to mono-nucleosome reads, and the number of reads falling in universal DNase-I hypersensitive regions, promoter regions, enhancer regions, and called peak regions. To compute these, we used putative promoter, enhancer, and DHS annotations from 127 cell types and tissues from the Roadmap Epigenomics Project. These annotations are provided in the flagship Roadmap Epigenomics Project publication^31^ and are available from the supplementary website http://compbio.mit.edu/roadmap in the ‘DNase-I accessible regulatory regions’ section. In brief, DNase-seq based chromatin accessible regions were labeled as promoter or enhancer based on chromatin state maps learned using 5 core histone modifications across the 127 cell types and tissues.

The graphs in Fig. 3 were taken from the output of the ATAqC pipeline for one representative sample (lateral mesoderm). The full set of graphs for all of our samples can be found in [Data Citation 5].

### High-throughput surface marker screening

**Figure 4:**
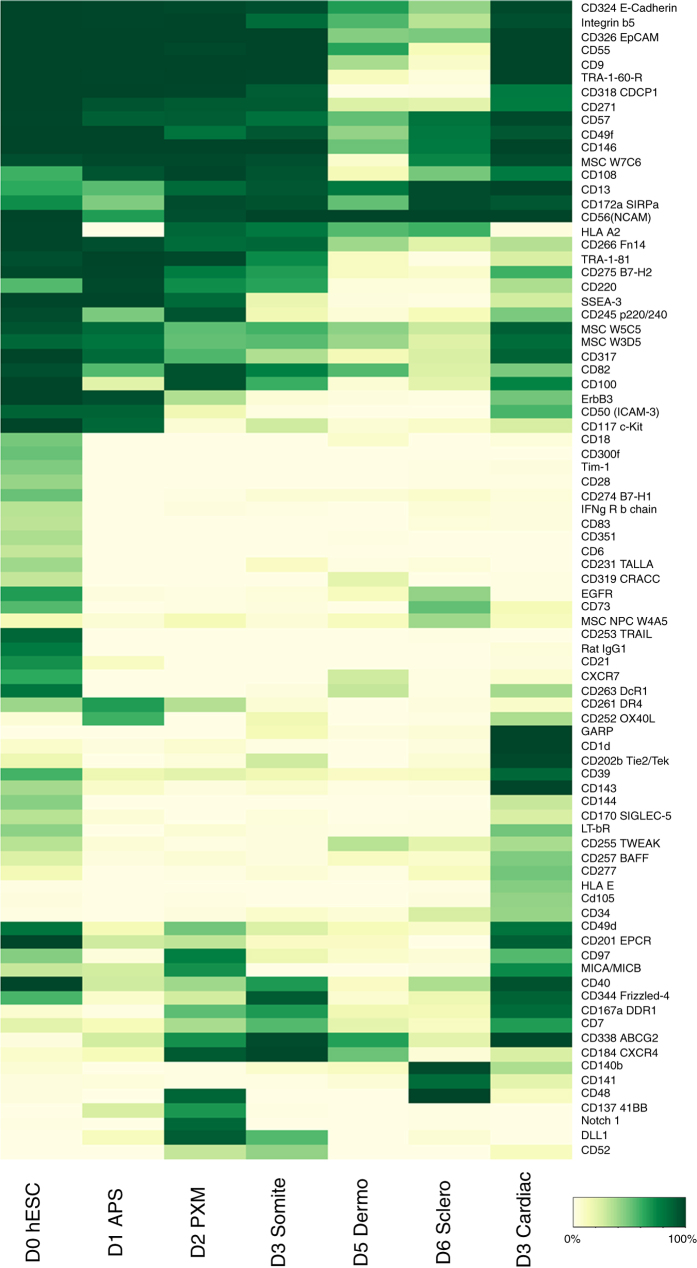
High-throughput surface marker screening. We show here a heatmap of all surface markers whose expression varied considerably across cell types, filtering out markers where less than 30% or more than 70% of cells across all cell types expressed the marker. The % refers to the percentage of cells of a given type that expressed a marker.

For validation, we focused on surface markers with lineage-specific expression (Fig. 4), and chose two surface markers for in-depth *in vivo* and *in vitro* validation: DLL1 (a marker of paraxial mesoderm) and GARP (a marker of cardiac mesoderm). *In situ* hybridization of zebrafish homologs of these genes (*deltaC*, the homolog of human DLL1 and *lrrc32*, the homolog of human GARP) was conducted in zebrafish embryos, which revealed fairly specific expression of *deltaC* in paraxial mesoderm and that of *lrrc32* in the developing heart tube *in vivo*^8^. Additionally, fluorescence-activated cell sorting (FACS) of DLL1+ cells from hESC-derived day 2 paraxial mesoderm cultures followed by bulk-population and single-cell RNA-seq revealed that all DLL1+ cells essentially expressed paraxial mesoderm transcription factors at the single-cell level^8^. Collectively, this reaffirmed that DLL1 and GARP respectively mark human paraxial and cardiac mesoderm.

### Differentiation

Our related publication^8^ focused on establishing the identity and function of the derived cell types, and we refer readers interested in those details to that manuscript. In brief, we verified cellular function through *in vivo* transplantation experiments and we assessed cellular identity and purity through molecular analyses of marker expression (RNA-seq, ATAC-seq, immunostaining, and flow cytometry).

On the molecular side, for each cell type we identified archetypic genes and surface markers based on biological knowledge and prior literature. We confirmed that the key genes were expressed at a population level through bulk RNA-seq and qPCR; then, through single-cell RNA-seq, immunostaining, or flow cytometry, we verified that the population was suitably homogeneous for those genes and surface markers^8^. On the basis of those metrics, the cell populations we derived were generally between 80 and 99% pure. Motif enrichment analysis of the open chromatin regions in each cell type (as measured by ATAC-seq) also yielded results consistent with their cellular identity, e.g., GATA motifs were significantly enriched in lateral and cardiac mesoderm.

We conducted transplantation experiments in immunodeficient mice to further verify the function of two human mesodermal cell-types derived from our differentiation process, namely sclerotome and cardiac mesoderm^8^. Sclerotome cells subcutaneously injected into immunodeficient mice self-organized to form an ectopic human bone, undergoing ossification, displaying spatial structure expected of human bone, and even attracting and becoming vascularized by mouse blood vessels. For the cardiac mesoderm, we first further differentiated them into cardiomyocytes through WNT blockade and BMP inhibition for four days and engineered them to express a constitutively-expressed *luciferase* reporter gene. To further test the functionality of these ESC-derived human cardiomyocytes, we developed an experimental system wherein ventricular fragments from week 15–17 human fetal heart^32^ were subcutaneously implanted into the mouse ear. We then transplanted ESC-derived cardiomyocytes directly into the human fetal heart graft and found that they engrafted the human heart tissue for at least 10 weeks, as measured by bioluminescence imaging of *luciferase*-expressing cardiomyocytes *in vivo*.

## Usage Notes

Researchers studying the single-cell RNA-seq data reported herein should note that inferences made from global comparisons (e.g., PCA or clustering) may be limited by experimental design, as each individual cell-type was processed on a separate Fluidigm C1 chip. Hence when comparing single-cell RNA-seq data from different cell-types it is difficult to account for batch effects arising from different chips. Our analysis, including comparisons of key lineage marker genes known to vary between distinct cell lineages, shows that the data are still valid. However, care should be taken in global comparisons that involve aggregating large numbers of genes, as the noise from batch effects could be substantial in that context; the bulk-population RNA-seq data could be used to verify results from such comparisons.

In our related publication^8^, we applied principal component analysis to the single-cell RNA-seq data to reconstruct the differentiation trajectory of paraxial mesoderm, somitomeres, and early somites in ‘pseudotime’, a concept first introduced in the context of single-cell RNA-seq by other groups (ref. 10 and ref. 33). Interested readers might want to apply these, and other more sophisticated trajectory reconstruction methods, to our single-cell data.

The variance in data quality across the ATAC-seq experiments, due to technical reasons (e.g., different numbers of starting reads or varying cell lysis) and biological reasons (e.g., distinct cell types may have different amounts of open chromatin), mean that care must also be taken when conducting global comparisons of ATAC-seq data. We found that rank-normalization (or, at one extreme, binarization) makes it easier to compare ATAC-seq data across cell-types, as opposed to using P-values, local IDR values, or measures of signal intensity to score each peak. Sub-sampling reads before peak-calling, as we did in our analysis, should only be done for global comparisons; researchers who are doing an in-depth study of one cell type should use all available reads that pass the filtering criteria for maximal information.

We are currently using this dataset to study the temporal changes in alternative splicing and long non-coding RNA expression as differentiation progresses. In addition, we are actively exploring the expression of repeat elements, including dormant retrotransposons, interspersed nuclear elements, Alu elements, and human endogenous retrovirus elements that may have a role in early human embryonic development. Readers who are interested in similar questions are welcome to contact us to discuss methods and collaborations.

## Additional Information

**How to cite this article:** Koh, P. W. *et al.* An atlas of transcriptional, chromatin accessibility, and surface marker changes in human mesoderm development. *Sci. Data* 3:160109 doi: 10.1038/sdata.2016.109 (2016).

**Publisher’s note:** Springer Nature remains neutral with regard to jurisdictional claims in published maps and institutional affiliations.

## Supplementary Material



## Figures and Tables

**Table 1:** Overall experimental metadata briefly describing each of the data sets available, with links to the appropriate data repository

**Source (Cell type)**	**Sample ID**	**Protocol 1**	**Protocol 2**	**Protocol 3**	**Data**
D0 hESC	H7_hESC_ATAC1	Nuclei isolation	ATAC-seq		SRR3689759
D0 hESC	H7_hESC_ATAC2	Nuclei isolation	ATAC-seq		SRR3689760
D0 hESC	H7hESC_1	RNA extraction	Bulk RNA-seq		SRR3439477
D0 hESC	H7hESC_2	RNA extraction	Bulk RNA-seq		SRR3439478
D0 hESC	H7hESC_3	RNA extraction	Bulk RNA-seq		SRR3439480
D0 hESC	H7Trzl	RNA extraction	Bulk RNA-seq		SRR3439481
D0 hESC	multiple	Single-cell capture	Single-cell RNA-seq		SRX1977195
D0 hESC	H7 hESC	Surface marker screening			10.6084/m9.figshare.3505817
D0 hESC	(processed peak calls)	Nuclei isolation	ATAC-seq		GSM2257291
D1 Anterior Primitive Streak	APS_ATAC3	Differentiation	Nuclei isolation	ATAC-seq	SRR3689761
D1 Anterior Primitive Streak	APS_ATAC4	Differentiation	Nuclei isolation	ATAC-seq	SRR3689762
D1 Anterior Primitive Streak	APS_1	Differentiation	RNA extraction	Bulk RNA-seq	SRR3439429
D1 Anterior Primitive Streak	APS_2	Differentiation	RNA extraction	Bulk RNA-seq	SRR3439430
D1 Anterior Primitive Streak	APS_3	Differentiation	RNA extraction	Bulk RNA-seq	SRR3439431
D1 Anterior Primitive Streak	multiple	Differentiation	Single-cell capture	Single-cell RNA-seq	SRX1977196
D1 Anterior Primitive Streak	Ant Primitive Streak (MIXL1-GFP+)	Differentiation	Surface marker screening		10.6084/m9.figshare.3505817
D1 Anterior Primitive Streak	(processed peak calls)	Differentiation	Nuclei isolation	ATAC-seq	GSM2257292
D1 Mid Primitive Streak	MPS_ATAC5	Differentiation	Nuclei isolation	ATAC-seq	SRR3689763
D1 Mid Primitive Streak	MPS_ATAC6	Differentiation	Nuclei isolation	ATAC-seq	SRR3689764
D1 Mid Primitive Streak	MPS_1	Differentiation	RNA extraction	Bulk RNA-seq	SRR3439482
D1 Mid Primitive Streak	MPS_2	Differentiation	RNA extraction	Bulk RNA-seq	SRR3439485
D1 Mid Primitive Streak	MPS_3	Differentiation	RNA extraction	Bulk RNA-seq	SRR3439486
D1 Mid Primitive Streak	MPS_4	Differentiation	RNA extraction	Bulk RNA-seq	SRR3439487
D1 Mid Primitive Streak	multiple	Differentiation	Single-cell capture	Single-cell RNA-seq	SRX1977197
D1 Mid Primitive Streak	(processed peak calls)	Differentiation	Nuclei isolation	ATAC-seq	GSM2257293
D2 DLL1− Paraxial Mesoderm	DLL1nD2nonPXM_1	Differentiation	RNA extraction	Bulk RNA-seq	SRR3439468
D2 DLL1− Paraxial Mesoderm	DLL1nD2nonPXM_2	Differentiation	RNA extraction	Bulk RNA-seq	SRR3439469
D2 DLL1+ Paraxial Mesoderm	DLL1pPXm_ATAC7	Differentiation	Nuclei isolation	ATAC-seq	SRR3689781
D2 DLL1+ Paraxial Mesoderm	DLL1pPXm_ATAC8	Differentiation	Nuclei isolation	ATAC-seq	SRR3689915
D2 DLL1+ Paraxial Mesoderm	DLL1pPXM_3	Differentiation	RNA extraction	Bulk RNA-seq	SRR3439471
D2 DLL1+ Paraxial Mesoderm	multiple	Differentiation	Single-cell capture	Single-cell RNA-seq	SRX1977198
D2 DLL1+ Paraxial Mesoderm	DLLpPXM_1	Differentiation	RNA extraction	Bulk RNA-seq	SRR3439472
D2 DLL1+ Paraxial Mesoderm	DLL1pPXM_2	Differentiation	RNA extraction	Bulk RNA-seq	SRR3439470
D2 DLL1+ Paraxial Mesoderm	(processed peak calls)	Differentiation	Nuclei isolation	ATAC-seq	GSM2257294
D2 Lateral Mesoderm	D2Ltm_ATAC10	Differentiation	Nuclei isolation	ATAC-seq	SRR3689918
D2 Lateral Mesoderm	D2Ltm_ATAC9	Differentiation	Nuclei isolation	ATAC-seq	SRR3689916
D2 Lateral Mesoderm	D2LtM_1	Differentiation	RNA extraction	Bulk RNA-seq	SRR3439434
D2 Lateral Mesoderm	D2LtM_2	Differentiation	RNA extraction	Bulk RNA-seq	SRR3439437
D2 Lateral Mesoderm	multiple	Differentiation	Single-cell capture	Single-cell RNA-seq	SRX1977202
D2 Lateral Mesoderm	(processed peak calls)	Differentiation	Nuclei isolation	ATAC-seq	GSM2257298
D2 Paraxial Mesoderm	Paraxial Mesoderm	Differentiation	Surface marker screening		10.6084/m9.figshare.3505817
D2.25 Somitomeres	Smtmrs_ATAC21	Differentiation	Nuclei isolation	ATAC-seq	SRR3689991
D2.25 Somitomeres	Smtmrs_ATAC22	Differentiation	Nuclei isolation	ATAC-seq	SRR3690220
D2.25 Somitomeres	multiple	Differentiation	Single-cell capture	Single-cell RNA-seq	SRX1977204
D2.25 Somitomeres	(processed peak calls)	Differentiation	Nuclei isolation	ATAC-seq	GSM2257300
D3 Cardiac Mesoderm	Cardiac Mesoderm (NKX2.5-GFP+)	Differentiation	Surface marker screening		10.6084/m9.figshare.3505817
D3 Early Somite	ESMT_ATAC13	Differentiation	Nuclei isolation	ATAC-seq	SRR3689931
D3 Early Somite	ESMT_ATAC14	Differentiation	Nuclei isolation	ATAC-seq	SRR3689932
D3 Early Somite	Smt_1	Differentiation	RNA extraction	Bulk RNA-seq	SRR3439490
D3 Early Somite	Smt_2	Differentiation	RNA extraction	Bulk RNA-seq	SRR3439491
D3 Early Somite	D3EarlySmt_1	Differentiation	RNA extraction	Bulk RNA-seq	SRR3439438
D3 Early Somite	D3EarlySmt_2	Differentiation	RNA extraction	Bulk RNA-seq	SRR3439440
D3 Early Somite	multiple	Differentiation	Single-cell capture	Single-cell RNA-seq	SRX1977199
D3 Early Somite	Smt_4	Differentiation	RNA extraction	Bulk RNA-seq	SRR3439494
D3 Early Somite	Smt_3	Differentiation	RNA extraction	Bulk RNA-seq	SRR3439493
D3 Early Somite	Early Somite	Differentiation	Surface marker screening		10.6084/m9.figshare.3505817
D3 Early Somite	(processed peak calls)	Differentiation	Nuclei isolation	ATAC-seq	GSM2257295
D3 GARP+ Cardiac Mesoderm	D3CrdcM_ATAC15	Differentiation	Nuclei isolation	ATAC-seq	SRR3689933
D3 GARP+ Cardiac Mesoderm	D3CrdcM_ATAC16	Differentiation	Nuclei isolation	ATAC-seq	SRR3689934
D3 GARP+ Cardiac Mesoderm	D3GARPpCrdcM_1	Differentiation	RNA extraction	Bulk RNA-seq	SRR3439441
D3 GARP+ Cardiac Mesoderm	D3GARPpCrdcM_2	Differentiation	RNA extraction	Bulk RNA-seq	SRR3439442
D3 GARP+ Cardiac Mesoderm	multiple	Differentiation	Single-cell capture	Single-cell RNA-seq	SRX1977203
D3 GARP+ Cardiac Mesoderm	(processed peak calls)	Differentiation	Nuclei isolation	ATAC-seq	GSM2257299
D5 Dermomyotome	Drmmtm_ATAC19	Differentiation	Nuclei isolation	ATAC-seq	SRR3689935
D5 Dermomyotome	Drmmtm_ATAC20	Differentiation	Nuclei isolation	ATAC-seq	SRR3689936
D5 Dermomyotome	Drmmtm_1	Differentiation	RNA extraction	Bulk RNA-seq	SRR3439474
D5 Dermomyotome	Drmmtm_2	Differentiation	RNA extraction	Bulk RNA-seq	SRR3439475
D5 Dermomyotome	D5CentralDrmmtm	Differentiation	RNA extraction	Bulk RNA-seq	SRR3439443
D5 Dermomyotome	multiple	Differentiation	Single-cell capture	Single-cell RNA-seq	SRX1977201
D5 Dermomyotome	Drmmtm_3	Differentiation	RNA extraction	Bulk RNA-seq	SRR3439476
D5 Dermomyotome	Dermomyotome	Differentiation	Surface marker screening		10.6084/m9.figshare.3505817
D5 Dermomyotome	(processed peak calls)	Differentiation	Nuclei isolation	ATAC-seq	GSM2257297
D6 PDGFRA+ Sclerotome	D6Sclrtm_ATAC11	Differentiation	Nuclei isolation	ATAC-seq	SRR3689921
D6 PDGFRA+ Sclerotome	D6Sclrtm_ATAC12	Differentiation	Nuclei isolation	ATAC-seq	SRR3689923
D6 PDGFRA+ Sclerotome	Sclrtm_1	Differentiation	RNA extraction	Bulk RNA-seq	SRR3439488
D6 PDGFRA+ Sclerotome	Sclrtm_2	Differentiation	RNA extraction	Bulk RNA-seq	SRR3439489
D6 PDGFRA+ Sclerotome	D6PDGFRApSclrtm_1	Differentiation	RNA extraction	Bulk RNA-seq	SRR3439456
D6 PDGFRA+ Sclerotome	multiple	Differentiation	Single-cell capture	Single-cell RNA-seq	SRX1977200
D6 PDGFRA+ Sclerotome	(processed peak calls)	Differentiation	Nuclei isolation	ATAC-seq	GSM2257296
D6 Sclerotome	Sclerotome	Differentiation	Surface marker screening		10.6084/m9.figshare.3505817

**Table 2:** Bulk-population RNA-seq metadata and mapping statistics.

**Sample ID**	**Celltype**	**Batch**	**Number of uniquely mapped reads**	**Percentage of uniquely mapping reads**
H7hESC_1	D0 H7 hESC	1	29077933	77.64
H7hESC_2	D0 H7 hESC	1	32593810	72.39
H7hESC_3	D0 H7 hESC	1	30800393	74.51
H7Trzl	D0 H7 hESC	3	60399642	76.01
APS_1	D1 Anterior Primitive Streak	1	29444532	71.43
APS_2	D1 Anterior Primitive Streak	1	30585671	72.73
APS_3	D1 Anterior Primitive Streak	3	62559303	74.76
MPS_1	D1 Mid Primitive Streak	1	32195105	74.5
MPS_2	D1 Mid Primitive Streak	1	29668483	76.07
MPS_3	D1 Mid Primitive Streak	3	64236835	73.91
MPS_4	D1 Mid Primitive Streak	3	76385886	72.9
DLL1nD2nonPXM_1	D2 DLL1− Paraxial Mesoderm	2	28555523	64.46
DLL1nD2nonPXM_2	D2 DLL1− Paraxial Mesoderm	2	24999466	66.51
DLL1pPXM_3	D2 DLL1+ Paraxial Mesoderm	2	11948788	50.27
DLLpPXM_1	D2 DLL1+ Paraxial Mesoderm	2	29595403	73.74
DLL1pPXM_2	D2 DLL1+ Paraxial Mesoderm	2	25504394	71.61
D2LtM_1	D2 Lateral Mesoderm	3	79623847	72.3
D2LtM_2	D2 Lateral Mesoderm	3	85281213	70.89
D3GARPpCrdcM_1	D3 GARP+ Cardiac Mesoderm	3	87530457	69.6
D3GARPpCrdcM_2	D3 GARP+ Cardiac Mesoderm	3	94587346	74.36
Smt_1	D3 Somite	1	15272975	57.37
Smt_2	D3 Somite	1	24702016	60.63
D3EarlySmt_1	D3 Somite	3	60723730	65.19
D3EarlySmt_2	D3 Somite	3	64467176	64.97
Smt_4	D3 Somite	2	14235841	62.14
Smt_3	D3 Somite	2	20701016	62.06
Drmmtm_1	D5 Dermomyotome	1	32318123	73.43
Drmmtm_2	D5 Dermomyotome	1	18890789	63.49
D5CentralDrmmtm	D5 Dermomyotome	3	126799726	69.06
Drmmtm_3	D5 Dermomyotome	2	10583923	65.92
Sclrtm_1	D6 PDGFRA+ Sclerotome	1	20751549	58.61
Sclrtm_2	D6 PDGFRA+ Sclerotome	1	23117071	66.89
D6PDGFRApSclrtm_1	D6 PDGFRA+ Sclerotome	3	102352286	74.86

**Table 3:** Single-cell RNA-seq metadata and mapping statistics

**Sample ID**	**Number of uniquely mapped reads**	**Percentage of uniquely mapping reads**	**Included in analysis?**
APS-p1c10r1	1496268	81.21	TRUE
APS-p1c10r3	1635980	79.9	TRUE
APS-p1c10r4	1462666	79.58	TRUE
APS-p1c10r6	1015763	79.94	TRUE
APS-p1c10r7	1649241	80.35	TRUE
APS-p1c10r8	631285	79.63	FALSE
APS-p1c11r2	2006570	79.83	TRUE
APS-p1c11r3	1571051	80.36	TRUE
APS-p1c11r6	1595606	80.29	TRUE
APS-p1c11r7	1611080	80.05	TRUE
APS-p1c12r2	1910166	80.12	TRUE
APS-p1c12r3	1453044	81.01	TRUE
APS-p1c12r4	1662275	80.47	TRUE
APS-p1c12r5	1804505	80.55	TRUE
APS-p1c12r6	1810735	76.06	TRUE
APS-p1c12r7	2065268	79.42	TRUE
APS-p1c1r2	1727374	80.73	TRUE
APS-p1c1r5	1756430	80.18	TRUE
APS-p1c1r8	1569290	77.97	TRUE
APS-p1c2r1	885464	80.93	FALSE
APS-p1c2r2	1926022	80.48	TRUE
APS-p1c2r3	1941685	80.73	TRUE
APS-p1c2r4	1700490	79.24	TRUE
APS-p1c2r6	1614915	79.99	TRUE
APS-p1c2r7	543563	76.21	FALSE
APS-p1c3r1	1600354	81.01	TRUE
APS-p1c3r3	1101602	81.42	TRUE
APS-p1c3r4	1620729	80.95	TRUE
APS-p1c3r5	1809789	80.39	TRUE
APS-p1c3r6	1820410	80.26	TRUE
APS-p1c4r2	1870031	80.81	TRUE
APS-p1c4r5	1767798	79.67	TRUE
APS-p1c4r6	1525333	79.93	TRUE
APS-p1c4r7	1816027	80.58	TRUE
APS-p1c4r8	1821569	80.37	TRUE
APS-p1c5r1	1800248	81.67	TRUE
APS-p1c5r2	1891607	81.05	TRUE
APS-p1c5r3	1492013	80.99	TRUE
APS-p1c5r4	1730157	80.21	TRUE
APS-p1c5r7	1701332	80.69	TRUE
APS-p1c5r8	1812781	80.93	TRUE
APS-p1c6r2	2181845	80.94	TRUE
APS-p1c6r3	1308024	81.28	TRUE
APS-p1c6r4	1733343	80.84	TRUE
APS-p1c6r5	1421945	77.27	TRUE
APS-p1c6r6	1719273	80.1	TRUE
APS-p1c6r8	1911832	74.68	TRUE
APS-p1c7r2	1545812	81.26	TRUE
APS-p1c7r3	1524138	80.8	TRUE
APS-p1c7r4	1072955	80.44	TRUE
APS-p1c7r5	1526998	80.09	TRUE
APS-p1c7r6	1577051	79.48	TRUE
APS-p1c7r8	840333	76.82	FALSE
APS-p1c8r1	1787534	81.16	TRUE
APS-p1c8r3	1353928	80.91	TRUE
APS-p1c8r4	1607464	80.79	TRUE
APS-p1c8r5	1536405	80.24	TRUE
APS-p1c8r8	418551	72.48	FALSE
APS-p1c9r1	1406401	80.75	TRUE
APS-p1c9r2	1706509	81.23	TRUE
APS-p1c9r3	1194409	81.22	TRUE
APS-p1c9r4	1679021	80.14	TRUE
APS-p1c9r6	1484868	80.4	TRUE
APS-p1c9r7	1760868	79.9	TRUE
cDM-p4c10r1	2019689	81.41	TRUE
cDM-p4c10r2	1965420	80.01	TRUE
cDM-p4c10r4	1870848	81.12	TRUE
cDM-p4c10r5	1884639	81.67	TRUE
cDM-p4c10r6	1847885	81.15	TRUE
cDM-p4c11r1	1911203	79.96	TRUE
cDM-p4c11r3	1717116	81.56	TRUE
cDM-p4c11r5	1662303	80.64	TRUE
cDM-p4c11r7	2072860	80.03	TRUE
cDM-p4c11r8	1910380	81.22	TRUE
cDM-p4c12r2	1915054	80.57	TRUE
cDM-p4c12r3	1933069	79.73	TRUE
cDM-p4c12r4	1817113	81.03	TRUE
cDM-p4c12r5	1996977	81.25	TRUE
cDM-p4c12r6	2111421	82.11	TRUE
cDM-p4c12r7	2230099	79.93	TRUE
cDM-p4c1r1	1828075	79.63	TRUE
cDM-p4c1r2	1994440	80.47	TRUE
cDM-p4c1r3	1984577	80.11	TRUE
cDM-p4c1r4	1978378	81.57	TRUE
cDM-p4c1r5	1894520	80.67	TRUE
cDM-p4c1r7	2191642	80.94	TRUE
cDM-p4c1r8	1889495	79.06	TRUE
cDM-p4c2r2	1838478	79.98	TRUE
cDM-p4c2r3	1829101	80.64	TRUE
cDM-p4c2r4	1876122	80.46	TRUE
cDM-p4c2r5	1757262	80.67	TRUE
cDM-p4c2r6	1779703	80.34	TRUE
cDM-p4c2r7	2062879	81.3	TRUE
cDM-p4c3r1	1904076	80.17	TRUE
cDM-p4c3r3	1995857	78.14	TRUE
cDM-p4c3r6	1762614	80.09	TRUE
cDM-p4c3r8	2011670	80.68	TRUE
cDM-p4c4r1	1746007	80.77	TRUE
cDM-p4c4r2	1748403	80.22	TRUE
cDM-p4c4r3	1765581	81.91	TRUE
cDM-p4c4r4	1741191	79.69	TRUE
cDM-p4c4r5	1817639	80.9	TRUE
cDM-p4c4r6	1655302	79.89	TRUE
cDM-p4c4r8	2141525	80.15	TRUE
cDM-p4c5r2	1932752	80.97	TRUE
cDM-p4c5r3	1829667	79.91	TRUE
cDM-p4c5r6	1657866	81.54	TRUE
cDM-p4c5r7	1936430	78.26	TRUE
cDM-p4c6r2	1862491	80.72	TRUE
cDM-p4c6r3	1869951	81.21	TRUE
cDM-p4c6r7	1993252	77.67	TRUE
cDM-p4c7r1	1788721	80.26	TRUE
cDM-p4c7r3	1745311	81.19	TRUE
cDM-p4c7r4	1583398	80.37	TRUE
cDM-p4c7r5	1867644	80.28	TRUE
cDM-p4c7r6	1437072	82.28	TRUE
cDM-p4c7r7	1973276	81.6	TRUE
cDM-p4c7r8	2539638	80.51	TRUE
cDM-p4c8r1	2232466	80.51	TRUE
cDM-p4c8r2	1924463	80.94	TRUE
cDM-p4c8r3	1966729	79.69	TRUE
cDM-p4c8r4	1833986	81.25	TRUE
cDM-p4c8r6	1739467	81.13	TRUE
cDM-p4c8r7	1702524	80.64	TRUE
cDM-p4c9r1	2089258	80.94	TRUE
cDM-p4c9r2	1599948	79.86	TRUE
cDM-p4c9r3	1817174	79.3	TRUE
cDM-p4c9r4	1992609	80.64	TRUE
cDM-p4c9r5	1983037	80.94	TRUE
cDM-p4c9r6	1726058	81.48	TRUE
cDM-p4c9r7	1961458	81.39	TRUE
D2_25somitomere-p9c10r2	1523221	83.23	TRUE
D2_25somitomere-p9c10r3	1281308	82.57	TRUE
D2_25somitomere-p9c10r4	1169199	82.93	TRUE
D2_25somitomere-p9c10r5	40654	78.75	FALSE
D2_25somitomere-p9c10r6	1489521	83.89	TRUE
D2_25somitomere-p9c10r7	1493339	82.92	TRUE
D2_25somitomere-p9c10r8	1351704	83.22	TRUE
D2_25somitomere-p9c11r1	1014330	83.67	TRUE
D2_25somitomere-p9c11r2	1444322	83.34	TRUE
D2_25somitomere-p9c11r3	1209061	81.38	TRUE
D2_25somitomere-p9c11r4	1236037	82.49	TRUE
D2_25somitomere-p9c11r6	1422020	81.21	TRUE
D2_25somitomere-p9c11r7	1519685	83.89	TRUE
D2_25somitomere-p9c11r8	1437974	83.12	TRUE
D2_25somitomere-p9c12r1	1123428	82.9	TRUE
D2_25somitomere-p9c12r2	1249940	82.57	TRUE
D2_25somitomere-p9c12r3	1092531	81.6	TRUE
D2_25somitomere-p9c12r4	1323472	83.09	TRUE
D2_25somitomere-p9c12r5	720869	79.78	FALSE
D2_25somitomere-p9c12r6	1464125	83.25	TRUE
D2_25somitomere-p9c12r7	1381278	83.13	TRUE
D2_25somitomere-p9c1r1	1258841	82.63	TRUE
D2_25somitomere-p9c1r2	1361435	83.28	TRUE
D2_25somitomere-p9c1r3	1072989	83.87	TRUE
D2_25somitomere-p9c1r5	1142438	83.3	TRUE
D2_25somitomere-p9c1r7	1571532	83.97	TRUE
D2_25somitomere-p9c2r1	1392293	83.59	TRUE
D2_25somitomere-p9c2r2	1381840	82.08	TRUE
D2_25somitomere-p9c2r3	861177	81.35	FALSE
D2_25somitomere-p9c2r4	1255387	83.14	TRUE
D2_25somitomere-p9c2r5	1126615	82.97	TRUE
D2_25somitomere-p9c2r6	1184860	82.84	TRUE
D2_25somitomere-p9c2r7	1363339	82.25	TRUE
D2_25somitomere-p9c2r8	1132088	83.75	TRUE
D2_25somitomere-p9c3r1	1124784	79.67	TRUE
D2_25somitomere-p9c3r2	1419998	83.21	TRUE
D2_25somitomere-p9c3r3	663489	81.92	FALSE
D2_25somitomere-p9c3r4	1256006	84.04	TRUE
D2_25somitomere-p9c3r5	871952	81.84	FALSE
D2_25somitomere-p9c3r6	1200864	81.86	TRUE
D2_25somitomere-p9c4r1	1444673	83.59	TRUE
D2_25somitomere-p9c4r2	1610883	82.27	TRUE
D2_25somitomere-p9c4r3	1297370	81.6	TRUE
D2_25somitomere-p9c4r4	1343121	82.28	TRUE
D2_25somitomere-p9c4r5	1572373	82.5	TRUE
D2_25somitomere-p9c4r6	1847242	81.79	TRUE
D2_25somitomere-p9c4r7	1350912	82.4	TRUE
D2_25somitomere-p9c5r1	1412254	82.56	TRUE
D2_25somitomere-p9c5r2	1548146	81.79	TRUE
D2_25somitomere-p9c5r3	1136419	80.33	TRUE
D2_25somitomere-p9c5r4	1237110	82.51	TRUE
D2_25somitomere-p9c5r5	1461058	82.54	TRUE
D2_25somitomere-p9c5r6	1528959	83.1	TRUE
D2_25somitomere-p9c5r7	1611756	83.15	TRUE
D2_25somitomere-p9c5r8	1349367	83.32	TRUE
D2_25somitomere-p9c6r1	1050349	83.15	TRUE
D2_25somitomere-p9c6r2	839879	82.34	FALSE
D2_25somitomere-p9c6r3	1105600	81.23	TRUE
D2_25somitomere-p9c6r4	914458	83.24	FALSE
D2_25somitomere-p9c6r5	1326280	83.65	TRUE
D2_25somitomere-p9c6r6	1204228	80.85	TRUE
D2_25somitomere-p9c6r7	1024907	80.51	TRUE
D2_25somitomere-p9c6r8	993224	81.11	FALSE
D2_25somitomere-p9c7r1	1147234	82.9	TRUE
D2_25somitomere-p9c7r2	496473	82.5	FALSE
D2_25somitomere-p9c7r3	1473842	82.22	TRUE
D2_25somitomere-p9c7r4	888574	80.67	FALSE
D2_25somitomere-p9c7r5	1187268	83.82	TRUE
D2_25somitomere-p9c7r6	1196961	82.93	TRUE
D2_25somitomere-p9c7r7	1311356	82.88	TRUE
D2_25somitomere-p9c7r8	1573973	80.19	TRUE
D2_25somitomere-p9c8r1	1596476	80.48	TRUE
D2_25somitomere-p9c8r2	1004266	82.24	TRUE
D2_25somitomere-p9c8r3	1259905	80.02	TRUE
D2_25somitomere-p9c8r4	729526	79.27	FALSE
D2_25somitomere-p9c8r5	1457363	83.58	TRUE
D2_25somitomere-p9c8r6	1550426	82.39	TRUE
D2_25somitomere-p9c8r7	1400347	82.37	TRUE
D2_25somitomere-p9c8r8	1504859	82.74	TRUE
D2_25somitomere-p9c9r1	1100181	81.06	TRUE
D2_25somitomere-p9c9r2	1413423	82.7	TRUE
D2_25somitomere-p9c9r3	1304785	82.72	TRUE
D2_25somitomere-p9c9r4	1314260	82.47	TRUE
D2_25somitomere-p9c9r5	1235620	82.91	TRUE
D2_25somitomere-p9c9r6	1486911	82.98	TRUE
D2_25somitomere-p9c9r7	1338839	82.78	TRUE
D2_25somitomere-p9c9r8	1379544	83.4	TRUE
DLL1PXM-p8c10r1	3999241	75.5	TRUE
DLL1PXM-p8c10r2	3788265	75.88	TRUE
DLL1PXM-p8c10r3	2031405	79.22	TRUE
DLL1PXM-p8c10r4	1325684	81.3	TRUE
DLL1PXM-p8c10r6	1205931	82.67	TRUE
DLL1PXM-p8c10r7	1366471	82.66	TRUE
DLL1PXM-p8c10r8	1375044	82.09	TRUE
DLL1PXM-p8c11r2	3714947	74.72	TRUE
DLL1PXM-p8c11r3	1981586	79.89	TRUE
DLL1PXM-p8c11r4	1319333	81.84	TRUE
DLL1PXM-p8c11r5	1232579	82.76	TRUE
DLL1PXM-p8c11r7	1390915	82.28	TRUE
DLL1PXM-p8c11r8	1401231	82.85	TRUE
DLL1PXM-p8c12r2	2980869	75.57	TRUE
DLL1PXM-p8c12r3	1429174	81.76	TRUE
DLL1PXM-p8c12r4	1208221	82.74	TRUE
DLL1PXM-p8c12r5	1252460	81.07	TRUE
DLL1PXM-p8c12r6	906562	82.4	FALSE
DLL1PXM-p8c1r1	3526553	75.99	TRUE
DLL1PXM-p8c1r2	3325434	76.19	TRUE
DLL1PXM-p8c1r3	1078123	79.54	TRUE
DLL1PXM-p8c1r4	1643660	80.34	TRUE
DLL1PXM-p8c1r6	1399270	81.6	TRUE
DLL1PXM-p8c1r7	1239893	83.18	TRUE
DLL1PXM-p8c1r8	1237398	82.31	TRUE
DLL1PXM-p8c2r1	3629648	76.36	TRUE
DLL1PXM-p8c2r2	3978623	73.86	TRUE
DLL1PXM-p8c2r3	1577386	76.8	TRUE
DLL1PXM-p8c2r4	1582398	81.74	TRUE
DLL1PXM-p8c2r5	1156589	82.48	TRUE
DLL1PXM-p8c2r6	1361632	83.53	TRUE
DLL1PXM-p8c2r7	1264376	83.2	TRUE
DLL1PXM-p8c3r1	3728869	74.18	TRUE
DLL1PXM-p8c3r2	4131313	74.81	TRUE
DLL1PXM-p8c3r3	1310539	81.06	TRUE
DLL1PXM-p8c3r4	2148413	79.67	TRUE
DLL1PXM-p8c3r6	901761	80.09	FALSE
DLL1PXM-p8c3r8	481938	79.15	FALSE
DLL1PXM-p8c4r1	1061118	79.8	TRUE
DLL1PXM-p8c4r3	2194501	79.64	TRUE
DLL1PXM-p8c4r4	1314632	82.26	TRUE
DLL1PXM-p8c4r5	1353872	82.44	TRUE
DLL1PXM-p8c4r6	549267	77.18	FALSE
DLL1PXM-p8c4r8	1037181	83.18	TRUE
DLL1PXM-p8c5r1	2785354	72.09	TRUE
DLL1PXM-p8c5r2	4124086	75.87	TRUE
DLL1PXM-p8c5r3	1662736	81.18	TRUE
DLL1PXM-p8c5r4	1160772	83.29	TRUE
DLL1PXM-p8c5r5	1171809	82.23	TRUE
DLL1PXM-p8c5r6	976060	82.23	FALSE
DLL1PXM-p8c5r8	1018066	82.28	TRUE
DLL1PXM-p8c6r1	2983359	66.13	FALSE
DLL1PXM-p8c6r3	1487686	80.86	TRUE
DLL1PXM-p8c6r4	604655	77.84	FALSE
DLL1PXM-p8c6r5	1376485	82.34	TRUE
DLL1PXM-p8c6r6	1211696	83.04	TRUE
DLL1PXM-p8c6r7	1191425	81.53	TRUE
DLL1PXM-p8c7r1	3571845	70.49	TRUE
DLL1PXM-p8c7r2	3704175	71.41	TRUE
DLL1PXM-p8c7r3	3163571	76.32	TRUE
DLL1PXM-p8c7r4	1157615	82.54	TRUE
DLL1PXM-p8c7r7	1233164	82.7	TRUE
DLL1PXM-p8c7r8	1563606	81.47	TRUE
DLL1PXM-p8c8r1	3776149	72.95	TRUE
DLL1PXM-p8c8r2	3701594	74.95	TRUE
DLL1PXM-p8c8r3	999340	81.42	FALSE
DLL1PXM-p8c8r5	1384003	82.52	TRUE
DLL1PXM-p8c8r6	1446561	82.68	TRUE
DLL1PXM-p8c8r7	1164536	81.7	TRUE
DLL1PXM-p8c8r8	1546021	82.63	TRUE
DLL1PXM-p8c9r2	3624856	73.49	TRUE
DLL1PXM-p8c9r4	1043989	83.14	TRUE
DLL1PXM-p8c9r5	1351713	82.38	TRUE
DLL1PXM-p8c9r6	1210519	83.69	TRUE
DLL1PXM-p8c9r8	1339457	82.17	TRUE
Earlysomite-p10c10r1	774819	73.46	FALSE
Earlysomite-p10c10r2	1423274	80.76	TRUE
Earlysomite-p10c10r3	1407180	81.52	TRUE
Earlysomite-p10c10r5	1602963	81.25	TRUE
Earlysomite-p10c10r7	2145410	80.91	TRUE
Earlysomite-p10c10r8	787492	83.13	FALSE
Earlysomite-p10c11r1	1532609	82.03	TRUE
Earlysomite-p10c11r2	1673517	82.76	TRUE
Earlysomite-p10c11r3	1395270	80.9	TRUE
Earlysomite-p10c11r4	962242	83.59	FALSE
Earlysomite-p10c11r5	1137531	82.47	TRUE
Earlysomite-p10c11r6	1092418	79.58	TRUE
Earlysomite-p10c11r7	63482	77.21	FALSE
Earlysomite-p10c12r2	1391725	80.16	TRUE
Earlysomite-p10c12r3	842843	78.69	FALSE
Earlysomite-p10c12r4	1378920	80.65	TRUE
Earlysomite-p10c12r6	1217242	79.83	TRUE
Earlysomite-p10c2r4	962879	82.33	FALSE
Earlysomite-p10c2r8	1443360	80.14	TRUE
Earlysomite-p10c3r2	1067474	75.63	TRUE
Earlysomite-p10c3r3	1232462	81.96	TRUE
Earlysomite-p10c4r1	1423363	81.59	TRUE
Earlysomite-p10c4r2	1340161	81.07	TRUE
Earlysomite-p10c4r3	971375	82.38	FALSE
Earlysomite-p10c4r4	1709900	78.35	TRUE
Earlysomite-p10c4r5	1343614	82.21	TRUE
Earlysomite-p10c4r6	1662512	82.42	TRUE
Earlysomite-p10c4r7	64492	76.25	FALSE
Earlysomite-p10c4r8	1121158	81.28	TRUE
Earlysomite-p10c5r1	1082459	78.95	TRUE
Earlysomite-p10c5r4	968698	82.09	FALSE
Earlysomite-p10c5r5	1712134	82.37	TRUE
Earlysomite-p10c5r6	1462368	81.79	TRUE
Earlysomite-p10c5r7	1931446	80.42	TRUE
Earlysomite-p10c5r8	1512212	82.03	TRUE
Earlysomite-p10c6r1	1392445	81.23	TRUE
Earlysomite-p10c6r2	1218625	82.46	TRUE
Earlysomite-p10c6r3	1086105	82.44	TRUE
Earlysomite-p10c6r4	1358585	82.64	TRUE
Earlysomite-p10c6r5	1185549	83.27	TRUE
Earlysomite-p10c6r6	1100330	82.46	TRUE
Earlysomite-p10c6r7	1965721	81.13	TRUE
Earlysomite-p10c6r8	2028813	80.93	TRUE
Earlysomite-p10c7r1	1465126	80.68	TRUE
Earlysomite-p10c9r5	1314685	81.83	TRUE
GARP-p6c10r3	11454	77.13	FALSE
GARP-p6c10r7	25539	78.51	FALSE
GARP-p6c10r8	26958	78.53	FALSE
GARP-p6c11r1	11768	78.14	FALSE
GARP-p6c11r3	10349	77.35	FALSE
GARP-p6c11r6	27293	79.3	FALSE
GARP-p6c12r1	14280	77.94	FALSE
GARP-p6c12r2	12766	77.57	FALSE
GARP-p6c12r6	32501	78.95	FALSE
GARP-p6c1r1	11162	77.14	FALSE
GARP-p6c1r2	9803	76.78	FALSE
GARP-p6c1r3	11573	78.04	FALSE
GARP-p6c1r5	14135	78.16	FALSE
GARP-p6c1r8	33495	78.12	FALSE
GARP-p6c2r1	12558	77.71	FALSE
GARP-p6c2r3	11597	77.57	FALSE
GARP-p6c2r5	16336	78.32	FALSE
GARP-p6c2r6	12629	77.23	FALSE
GARP-p6c2r7	22792	78.73	FALSE
GARP-p6c3r1	15130	78.47	FALSE
GARP-p6c3r2	13706	77.69	FALSE
GARP-p6c3r4	14666	78.7	FALSE
GARP-p6c3r5	16248	78.41	FALSE
GARP-p6c3r7	29841	77.87	FALSE
GARP-p6c3r8	29254	78.35	FALSE
GARP-p6c4r4	17827	78.78	FALSE
GARP-p6c4r5	14303	77.57	FALSE
GARP-p6c4r7	25810	78.01	FALSE
GARP-p6c4r8	23401	77.92	FALSE
GARP-p6c5r2	11507	76.33	FALSE
GARP-p6c5r3	14371	77.13	FALSE
GARP-p6c5r5	14500	78.36	FALSE
GARP-p6c5r6	14749	77.74	FALSE
GARP-p6c5r7	30506	78.86	FALSE
GARP-p6c6r3	12627	76.86	FALSE
GARP-p6c6r6	17101	78.41	FALSE
GARP-p6c6r8	27028	78.21	FALSE
GARP-p6c7r1	12492	77.77	FALSE
GARP-p6c7r2	13090	77.52	FALSE
GARP-p6c7r3	10551	76.94	FALSE
GARP-p6c7r5	18259	78.01	FALSE
GARP-p6c7r6	14548	78.42	FALSE
GARP-p6c7r7	29833	78.89	FALSE
GARP-p6c8r1	11066	77.66	FALSE
GARP-p6c8r3	11671	76.37	FALSE
GARP-p6c8r4	12803	78.41	FALSE
GARP-p6c8r6	13057	78.01	FALSE
GARP-p6c8r7	26229	78.36	FALSE
GARP-p6c9r3	13597	77.83	FALSE
GARP-p6c9r4	16741	77.67	FALSE
GARP-p6c9r5	16141	77.49	FALSE
GARP-p6c9r8	32136	79.22	FALSE
H7hESC-p7c10r1	15212	77.81	FALSE
H7hESC-p7c10r2	13042	77.38	FALSE
H7hESC-p7c10r3	3040176	78.93	TRUE
H7hESC-p7c10r4	140876	69.78	FALSE
H7hESC-p7c10r5	2812947	79.05	TRUE
H7hESC-p7c10r7	831290	79.19	FALSE
H7hESC-p7c10r8	3601056	74.05	TRUE
H7hESC-p7c11r4	2607183	77.86	TRUE
H7hESC-p7c11r6	3000473	76	TRUE
H7hESC-p7c11r7	3363517	78.13	TRUE
H7hESC-p7c12r1	13410	77.49	FALSE
H7hESC-p7c12r2	12037	77.87	FALSE
H7hESC-p7c12r3	124096	71.79	FALSE
H7hESC-p7c12r4	104487	72.21	FALSE
H7hESC-p7c12r6	213692	50.66	FALSE
H7hESC-p7c12r7	3338221	74.7	TRUE
H7hESC-p7c1r1	24837	78.81	FALSE
H7hESC-p7c1r2	13352	77.58	FALSE
H7hESC-p7c1r3	129929	77.24	FALSE
H7hESC-p7c1r4	3110798	74.28	TRUE
H7hESC-p7c1r5	3155989	76.56	TRUE
H7hESC-p7c1r6	2792444	77.2	TRUE
H7hESC-p7c1r7	3344496	74.61	TRUE
H7hESC-p7c1r8	3511007	73.82	TRUE
H7hESC-p7c2r1	13618	78.42	FALSE
H7hESC-p7c2r3	2197303	72.9	TRUE
H7hESC-p7c2r4	3165871	79.03	TRUE
H7hESC-p7c2r5	3414200	73.2	TRUE
H7hESC-p7c2r6	2821648	75.67	TRUE
H7hESC-p7c2r7	2629633	75.39	TRUE
H7hESC-p7c2r8	3549143	75.69	TRUE
H7hESC-p7c3r1	16109	77.62	FALSE
H7hESC-p7c3r3	3107167	75.44	TRUE
H7hESC-p7c3r4	3200798	77.93	TRUE
H7hESC-p7c3r5	3046981	75.99	TRUE
H7hESC-p7c3r6	2052182	77.7	TRUE
H7hESC-p7c3r7	3104321	72.47	TRUE
H7hESC-p7c4r2	16580	77.12	FALSE
H7hESC-p7c4r3	3006452	78.27	TRUE
H7hESC-p7c4r6	566011	78.16	FALSE
H7hESC-p7c4r7	3183684	78.29	TRUE
H7hESC-p7c4r8	3285656	74.13	TRUE
H7hESC-p7c5r2	14747	77.51	FALSE
H7hESC-p7c5r3	3568139	77.64	TRUE
H7hESC-p7c5r4	2480859	79.42	TRUE
H7hESC-p7c5r5	2384075	78.8	TRUE
H7hESC-p7c5r6	936672	67.61	FALSE
H7hESC-p7c5r8	3519477	76.97	TRUE
H7hESC-p7c6r1	11228	77.48	FALSE
H7hESC-p7c6r2	12039	77.25	FALSE
H7hESC-p7c6r3	2926496	75.73	TRUE
H7hESC-p7c6r4	1258451	78.46	TRUE
H7hESC-p7c6r5	2606146	79.8	TRUE
H7hESC-p7c6r6	2387516	78.12	TRUE
H7hESC-p7c6r7	2876846	77.01	TRUE
H7hESC-p7c6r8	3687463	76.39	TRUE
H7hESC-p7c7r1	15548	77.71	FALSE
H7hESC-p7c7r3	3191548	78.21	TRUE
H7hESC-p7c7r5	2952936	78.61	TRUE
H7hESC-p7c7r6	3058342	77.42	TRUE
H7hESC-p7c7r7	2851034	77.88	TRUE
H7hESC-p7c7r8	3239712	75.89	TRUE
H7hESC-p7c8r1	13339	77.57	FALSE
H7hESC-p7c8r2	13234	77.22	FALSE
H7hESC-p7c8r3	3105874	73.86	TRUE
H7hESC-p7c8r5	3249837	76.01	TRUE
H7hESC-p7c8r6	3171979	77.12	TRUE
H7hESC-p7c8r7	2920896	74.23	TRUE
H7hESC-p7c8r8	3879315	74.84	TRUE
H7hESC-p7c9r1	15088	77.63	FALSE
H7hESC-p7c9r2	16111	79.54	FALSE
H7hESC-p7c9r3	2087164	79.75	TRUE
H7hESC-p7c9r5	2864608	78.63	TRUE
H7hESC-p7c9r6	2225023	78.87	TRUE
H7hESC-p7c9r7	2832054	78.63	TRUE
H7hESC-p7c9r8	3388549	75.62	TRUE
LatM-p3c10r1	1848645	80.26	TRUE
LatM-p3c10r3	1711542	78.22	TRUE
LatM-p3c10r6	1225845	82.13	TRUE
LatM-p3c11r1	2036629	80.18	TRUE
LatM-p3c11r3	1853196	80.2	TRUE
LatM-p3c11r4	1620883	80.38	TRUE
LatM-p3c11r5	1758473	79.52	TRUE
LatM-p3c11r8	1820674	80.73	TRUE
LatM-p3c12r2	1787205	81.83	TRUE
LatM-p3c12r4	1473145	81.97	TRUE
LatM-p3c12r5	1728367	80.65	TRUE
LatM-p3c12r7	1754434	80.59	TRUE
LatM-p3c1r1	1776278	81.49	TRUE
LatM-p3c1r4	1790186	81.03	TRUE
LatM-p3c1r6	1581544	80.21	TRUE
LatM-p3c1r7	1711082	80.03	TRUE
LatM-p3c2r1	1786408	80.57	TRUE
LatM-p3c2r2	1880860	80.72	TRUE
LatM-p3c2r3	1760165	80.96	TRUE
LatM-p3c2r5	1751354	80.27	TRUE
LatM-p3c2r6	1623023	80.2	TRUE
LatM-p3c2r7	1858784	79.49	TRUE
LatM-p3c3r2	1442559	79.79	TRUE
LatM-p3c3r8	1705278	80.16	TRUE
LatM-p3c4r1	1829541	80.95	TRUE
LatM-p3c4r2	1931289	81.29	TRUE
LatM-p3c4r4	1881071	80.69	TRUE
LatM-p3c4r5	1650957	78.83	TRUE
LatM-p3c4r6	1668589	80.08	TRUE
LatM-p3c4r7	1890628	79.95	TRUE
LatM-p3c4r8	1951557	79.25	TRUE
LatM-p3c5r1	1937785	81.57	TRUE
LatM-p3c5r2	1858567	79.88	TRUE
LatM-p3c5r4	1842332	79.91	TRUE
LatM-p3c5r6	1382254	81.5	TRUE
LatM-p3c5r8	1843866	81.03	TRUE
LatM-p3c6r1	1860437	78.79	TRUE
LatM-p3c6r4	1818224	80.47	TRUE
LatM-p3c6r7	2014636	79.86	TRUE
LatM-p3c6r8	1922478	79.64	TRUE
LatM-p3c7r1	1773998	81.46	TRUE
LatM-p3c7r2	1866162	80.69	TRUE
LatM-p3c7r3	1837113	81.17	TRUE
LatM-p3c7r5	1625469	79.99	TRUE
LatM-p3c7r6	1610642	79.42	TRUE
LatM-p3c8r2	1858425	80.39	TRUE
LatM-p3c8r3	1902659	80.99	TRUE
LatM-p3c8r7	2089988	78.8	TRUE
LatM-p3c8r8	1858528	80.44	TRUE
LatM-p3c9r1	1688880	82.07	TRUE
LatM-p3c9r2	1798283	81.47	TRUE
LatM-p3c9r3	1889540	79.88	TRUE
LatM-p3c9r4	1718310	80.47	TRUE
LatM-p3c9r5	1555638	79.93	TRUE
LatM-p3c9r7	1983700	79.74	TRUE
MPS3-p5c10r4	20381	76.7	FALSE
MPS3-p5c10r5	10555	78.21	FALSE
MPS3-p5c10r6	13522	78.05	FALSE
MPS3-p5c10r7	14699	77.16	FALSE
MPS3-p5c10r8	15635	76.64	FALSE
MPS3-p5c11r4	20558	76.27	FALSE
MPS3-p5c11r5	12339	78.61	FALSE
MPS3-p5c11r6	12125	77.2	FALSE
MPS3-p5c11r7	10559	77.66	FALSE
MPS3-p5c11r8	13773	77.09	FALSE
MPS3-p5c12r1	1948756	80.33	TRUE
MPS3-p5c12r2	22024	67.32	FALSE
MPS3-p5c12r3	20980	75.67	FALSE
MPS3-p5c12r4	2030771	80.97	TRUE
MPS3-p5c12r5	12577	78.13	FALSE
MPS3-p5c12r6	9270	76.62	FALSE
MPS3-p5c1r1	2477457	78.55	TRUE
MPS3-p5c1r3	2313945	80.07	TRUE
MPS3-p5c1r4	1637354	78.79	TRUE
MPS3-p5c1r7	10672	77.48	FALSE
MPS3-p5c2r1	2480808	79.2	TRUE
MPS3-p5c2r2	2263965	80.32	TRUE
MPS3-p5c2r3	2208614	79.38	TRUE
MPS3-p5c2r4	1672830	79.15	TRUE
MPS3-p5c2r5	2023398	80.15	TRUE
MPS3-p5c2r6	10750	77.89	FALSE
MPS3-p5c2r8	14327	76.66	FALSE
MPS3-p5c3r1	2012787	80.76	TRUE
MPS3-p5c3r8	12363	77.12	FALSE
MPS3-p5c4r1	25576	75.89	FALSE
MPS3-p5c4r2	1882352	81.27	TRUE
MPS3-p5c4r5	20462	77.17	FALSE
MPS3-p5c4r7	13299	78.95	FALSE
MPS3-p5c4r8	14242	78.48	FALSE
MPS3-p5c5r1	1405075	80.67	TRUE
MPS3-p5c5r2	2165346	79.87	TRUE
MPS3-p5c5r3	2332677	80.73	TRUE
MPS3-p5c5r5	10762	78.73	FALSE
MPS3-p5c6r1	2334672	78.86	TRUE
MPS3-p5c6r3	23901	77.09	FALSE
MPS3-p5c6r4	19745	75.9	FALSE
MPS3-p5c6r5	13045	78.17	FALSE
MPS3-p5c6r7	11005	77.97	FALSE
MPS3-p5c7r1	1675737	81.22	TRUE
MPS3-p5c7r2	2191692	79.27	TRUE
MPS3-p5c7r3	26703	76.97	FALSE
MPS3-p5c7r4	2077373	80.51	TRUE
MPS3-p5c7r8	14041	77.49	FALSE
MPS3-p5c8r1	1823770	81.26	TRUE
MPS3-p5c8r3	21160	77.06	FALSE
MPS3-p5c8r4	1841927	79.76	TRUE
MPS3-p5c8r5	8907	77.51	FALSE
MPS3-p5c8r7	9871	78.68	FALSE
MPS3-p5c8r8	12270	77.43	FALSE
MPS3-p5c9r3	24057	77.4	FALSE
MPS3-p5c9r4	2075554	80.11	TRUE
MPS3-p5c9r5	12517	77.45	FALSE
MPS3-p5c9r6	10794	78.06	FALSE
MPS3-p5c9r7	12506	78.54	FALSE
MPS3-p5c9r8	12816	77.5	FALSE
Sclerotome-p2c10r1	1802955	81.48	TRUE
Sclerotome-p2c10r2	1721023	80.74	TRUE
Sclerotome-p2c10r3	1800314	81.44	TRUE
Sclerotome-p2c10r4	2110665	79.43	TRUE
Sclerotome-p2c10r5	1882311	80.88	TRUE
Sclerotome-p2c10r6	1630387	80.23	TRUE
Sclerotome-p2c10r7	1845782	80.28	TRUE
Sclerotome-p2c10r8	1967016	80.1	TRUE
Sclerotome-p2c11r1	1970304	80.91	TRUE
Sclerotome-p2c11r3	1778989	80.41	TRUE
Sclerotome-p2c11r4	1782802	79.6	TRUE
Sclerotome-p2c11r6	1531583	80.34	TRUE
Sclerotome-p2c11r7	1577302	79.73	TRUE
Sclerotome-p2c12r1	24599	74.18	FALSE
Sclerotome-p2c12r3	1840700	80.81	TRUE
Sclerotome-p2c12r5	2083710	80.26	TRUE
Sclerotome-p2c1r1	1334198	80.66	TRUE
Sclerotome-p2c1r2	1943784	79.47	TRUE
Sclerotome-p2c1r4	1890414	80.28	TRUE
Sclerotome-p2c1r5	1912599	80.49	TRUE
Sclerotome-p2c1r6	1799140	81.08	TRUE
Sclerotome-p2c1r7	1777353	81.04	TRUE
Sclerotome-p2c2r2	1568544	81	TRUE
Sclerotome-p2c2r3	737599	81.34	FALSE
Sclerotome-p2c2r6	1919205	80.04	TRUE
Sclerotome-p2c2r8	973149	80.54	FALSE
Sclerotome-p2c3r1	1749624	81.93	TRUE
Sclerotome-p2c3r2	1908393	80.17	TRUE
Sclerotome-p2c3r3	1884627	80.5	TRUE
Sclerotome-p2c3r4	1635739	80.97	TRUE
Sclerotome-p2c3r6	1840941	81.35	TRUE
Sclerotome-p2c3r7	1801468	79.94	TRUE
Sclerotome-p2c3r8	1856378	80.14	TRUE
Sclerotome-p2c4r1	2033025	81.18	TRUE
Sclerotome-p2c4r3	1881229	80.21	TRUE
Sclerotome-p2c4r4	1733463	80.65	TRUE
Sclerotome-p2c4r5	1834616	79.73	TRUE
Sclerotome-p2c4r6	1414856	80.68	TRUE
Sclerotome-p2c4r7	1723879	79.71	TRUE
Sclerotome-p2c4r8	1784598	77.18	TRUE
Sclerotome-p2c5r1	1901144	81.28	TRUE
Sclerotome-p2c5r3	1785933	79.08	TRUE
Sclerotome-p2c5r4	1611140	77.72	TRUE
Sclerotome-p2c5r6	1570688	81.79	TRUE
Sclerotome-p2c5r7	1982820	80.76	TRUE
Sclerotome-p2c6r3	1809586	80.19	TRUE
Sclerotome-p2c6r5	1739098	78.21	TRUE
Sclerotome-p2c6r6	1660622	81.57	TRUE
Sclerotome-p2c6r7	1775201	79.93	TRUE
Sclerotome-p2c6r8	1835513	81.13	TRUE
Sclerotome-p2c7r1	1987120	81.32	TRUE
Sclerotome-p2c7r2	1347448	80.36	TRUE
Sclerotome-p2c7r3	1648535	80.24	TRUE
Sclerotome-p2c7r4	723428	80.49	FALSE
Sclerotome-p2c7r5	1850945	80.51	TRUE
Sclerotome-p2c7r6	1685250	78.77	TRUE
Sclerotome-p2c8r2	1592429	80.56	TRUE
Sclerotome-p2c8r3	1678306	80.05	TRUE
Sclerotome-p2c8r4	1162892	81.04	TRUE
Sclerotome-p2c8r5	1901339	81.21	TRUE
Sclerotome-p2c8r6	1731477	79.41	TRUE
Sclerotome-p2c8r7	1609297	78.87	TRUE
Sclerotome-p2c8r8	1742708	77.69	TRUE
Sclerotome-p2c9r1	1842849	81.26	TRUE
Sclerotome-p2c9r2	1858150	79.13	TRUE
Sclerotome-p2c9r3	1582883	81.39	TRUE
Sclerotome-p2c9r4	1782125	80.89	TRUE
Sclerotome-p2c9r5	1811432	79.69	TRUE
Sclerotome-p2c9r6	1709090	80.25	TRUE

**Table 4:** ATAC-seq metadata and quality control statistics

**Sample ID**	**Celltype**	**Date ATAC was performed**	**Date of library prep**	**Read count from sequencer**	**Read count successfully aligned**	**Read count after filtering for mapping quality**	**Read count after removing duplicate reads**	**Read count after removing mitochondrial reads**	**Non-Redundant Fraction (NRF)**	**PBC1**	**PBC2**	**Fraction of reads in NFR**	**NFR/mono-nuc reads**	**Presence of NFR peak**	**Presence of Mono-Nuc peak**	**Raw peaks**	**IDR peaks**	**TSS enrichment**	**Number of reads in universal DHS regions**	**Number of reads in promoter regions**	**Number of reads in enhancer regions**	**Number of reads in called peak regions**
H7_hESC_ATAC1	D0 hESC	11/8/2015	13/8/2015	93261696	89445943	63427355	48401487	33057240	0.874173	0.877391	7.963726	0.672145531	4.48815287	OK	OK	348998	72107	5.677532655	7501297	2181218	9420055	7113974
H7_hESC_ATAC2	D0 hESC	11/8/2015	13/8/2015	151825178	145741374	103141220	74914035	46080346	0.784357	0.780965	4.355791	0.594905988	3.111463078	OK	OK	254898	72107	5.417971046	8093722	2271676	10309049	6258218
APS_ATAC3	D1 APS	4/8/2015	13/8/2015	88401812	83952797	60558447	42312884	23641140	0.589525	0.549898	1.940211	0.591169991	3.182374684	OK	OK	345846	59125	6.454679332	5428052	1625595	6652426	5366166
APS_ATAC4	D1 APS	4/8/2015	13/8/2015	66716160	63482035	51529472	44238724	36710784	0.788187	0.778657	4.278458	0.647351136	4.050393391	OK	OK	243565	59125	5.615858894	7885350	2329280	10127389	6313436
MPS_ATAC5	D1 MPS	4/8/2015	13/8/2015	129125340	123708067	86733447	60812283	34369150	0.737118	0.727553	3.416704	0.594271523	3.19534847	OK	OK	359273	57945	6.99517666	8210077	2525384	9790941	8632701
MPS_ATAC6	D1 MPS	4/8/2015	13/8/2015	128802054	122229341	96453862	81570579	66255516	0.823556	0.818931	5.306797	0.678716288	4.711491841	OK	OK	258726	57945	4.580237486	7712954	2117515	10307394	5862319
DLL1pPXm_ATAC7	D2 DLL1+ PXM	6/8/2015	13/8/2015	191702808	183718196	132414659	99511940	66060614	0.870262	0.875024	7.831789	0.6619457	4.286335569	OK	OK	276391	109250	7.628156666	9726101	3070226	11125097	10862818
DLL1pPXm_ATAC8	D2 DLL1+ PXM	6/8/2015	13/8/2015	147291642	140788669	100167093	74377015	48138988	0.876385	0.882327	8.327058	0.648651946	4.078567409	OK	OK	277230	109250	6.923491809	9028293	2770033	10754812	9077103
D2Ltm_ATAC9	D2 LatM	13/8/2015	26/8/2015	95742814	89161027	55998269	38367024	20466238	0.809605	0.81417	5.168049	0.645042836	3.413532025	OK	OK	276175	83109	10.03275293	5519359	1819707	6117321	5451176
D2Ltm_ATAC10	D2 LatM	13/8/2015	26/8/2015	192134220	179733937	113070226	75469447	37284730	0.738965	0.735284	3.560357	0.618695962	3.008369035	OK	OK	271235	83109	9.294310086	9382874	3039301	10769509	9234041
D6Sclrtm_ATAC11	D6 Sclerotome	13/8/2015	26/8/2015	328044572	305242787	181293318	101400785	20559368	0.35659	0.275679	1.094047	0.620269839	3.299242828	OK	OK	253472	100590	17.34139067	6701318	3110272	5575866	9432698
D6Sclrtm_ATAC12	D6 Sclerotome	13/8/2015	26/8/2015	138904558	130498363	77296400	45578855	13471630	0.591614	0.570517	2.065099	0.622210362	3.106016386	OK	OK	273702	100590	22.34978045	5050293	2376784	3803650	7543825
ESMT_ATAC13	D3 Somite	10/8/2015	26/8/2015	97028570	90293961	63339679	50618923	37539350	0.898727	0.903776	10.279185	0.603838363	2.810326908	OK	OK	282813	116312	12.47182107	11055518	4184717	10999339	14172891
ESMT_ATAC14	D3 Somite	10/8/2015	26/8/2015	91342558	84065314	58377777	46807655	34919048	0.90416	0.90955	10.96564	0.63869863	3.207448669	OK	OK	269952	116312	11.90248274	10346635	3811873	10541840	12560254
D3CrdcM_ATAC15	D3 Cardiac	10/8/2015	26/8/2015	191778720	183897391	125066804	80344503	35114474	0.817896	0.842331	6.475016	0.657794431	3.578035603	OK	OK	319458	180380	20.14099152	15988868	5748762	13938902	26578992
D3CrdcM_ATAC16	D3 Cardiac	10/8/2015	26/8/2015	134871886	129649682	87780976	57145039	26151482	0.839505	0.863365	7.520129	0.670442227	3.796855645	OK	OK	286851	180380	19.13967334	11303563	4006843	10070805	16347057
Drmmtm_ATAC19	D5 Dermomytome	12/8/2015	26/8/2015	152978836	144566390	108536158	84652575	60077794	0.79836	0.805027	5.208383	0.701894281	5.092762813	OK	OK	263326	48916	8.862852148	8300267	2617001	10695931	7189919
Drmmtm_ATAC20	D5 Dermomytome	12/8/2015	26/8/2015	71382368	67502665	50296672	40140535	29655914	0.865341	0.871256	7.841101	0.688026099	4.642126486	OK	OK	285309	48916	8.873565459	6769259	2144352	8681073	5847726
Smtmrs_ATAC21	D2.25 Somitomere	6/8/2015	26/8/2015	425477876	407097668	310085861	238535286	165584332	0.791969	0.797997	4.973526	0.730157497	5.929894484	OK	OK	280874	83545	9.602687176	9101861	2824194	11178718	8781283
Smtmrs_ATAC22	D2.25 Somitomere	6/8/2015	26/8/2015	178465724	169037246	129385205	104336662	78489898	0.86589	0.870784	7.749138	0.70693823	4.987984435	OK	OK	277098	83545	9.442978111	9099713	2875813	11146695	9079702

**Table 5:** Surface marker screening metadata

**Well**	**Antigen**	**Antibody Clone**	**Antibody Type**	**Catalog No. (Biolegend)**
*LEGENDSCREEN PLATE #1*				
1	Blank			
A2	CD1a	HI149	Mouse IgG1, κ	300106
A3	CD1b	SN13 (K5- 1B8)	Mouse IgG1, κ	329108
A4	CD1c	L161	Mouse IgG1, κ	331506
A5	CD1d	51.1	Mouse IgG2b, κ	350306
A6	CD2	RPA-2.10	Mouse IgG1, κ	300208
A7	CD3	HIT3a	Mouse IgG2a, κ	300308
A8	CD4	RPA-T4	Mouse IgG1, κ	300508
A9	CD5	UCHT2	Mouse IgG1, κ	300608
A10	CD6	BL-CD6	Mouse IgG1, κ	313906
A11	CD7	CD7-6B7	Mouse IgG2a, κ	343106
A12	CD8a	HIT8a	Mouse IgG1, κ	300908
B1	CD9	HI9a	Mouse IgG1, κ	312106
B2	CD10	HI10a	Mouse IgG1, κ	312204
B3	CD11a	HI111	Mouse IgG1, κ	301208
B4	CD11b	ICRF44	Mouse IgG1, κ	301306
B5	CD11b (activated)	CBRM1/5	Mouse IgG1, κ	301406
B6	CD11c	3.9	Mouse IgG1, κ	301606
B7	CD13	WM15	Mouse IgG1, κ	301704
B8	CD14	M5E2	Mouse IgG2a, κ	301806
B9	CD15 (SSEA-1)	W6D3	Mouse IgG1, κ	323006
B10	CD16	3G8	Mouse IgG1, κ	302008
B11	CD18	TS1/18	Mouse IgG1, κ	302108
B12	CD19	HIB19	Mouse IgG1, κ	302208
C1	CD20	2H7	Mouse IgG2b, κ	302306
C2	CD21	Bu32	Mouse IgG1, κ	354904
C3	CD22	HIB22	Mouse IgG1, κ	302506
C4	CD23	EBVCS-5	Mouse IgG1, κ	338508
C5	CD24	ML5	Mouse IgG2a, κ	311106
C6	CD25	BC96	Mouse IgG1, κ	302606
C7	CD26	BA5b	Mouse IgG2a, κ	302706
C8	CD27	O323	Mouse IgG1, κ	302808
C9	CD28	CD28.2	Mouse IgG1, κ	302908
C10	CD29	TS2/16	Mouse IgG1, κ	303004
C11	CD30	BY88	Mouse IgG1, κ	333906
C12	CD31	WM59	Mouse IgG1, κ	303106
D1	CD32	FUN-2	Mouse IgG2b, κ	303206
D2	CD33	WM53	Mouse IgG1, κ	303404
D3	CD34	581	Mouse IgG1, κ	343506
D4	CD35	E11	Mouse IgG1, κ	333406
D5	CD36	5–271	Mouse IgG2a, κ	336206
D6	CD38	HIT2	Mouse IgG1, κ	303506
D7	CD39	A1	Mouse IgG1, κ	328208
D8	CD40	HB14	Mouse IgG1, κ	313006
D9	CD41	HIP8	Mouse IgG1, κ	303706
D10	CD42b	HIP1	Mouse IgG1, κ	303906
D11	CD43	CD43-10G7	Mouse IgG1, κ	343204
D12	CD44	BJ18	Mouse IgG1, κ	338808
E1	CD45	HI30	Mouse IgG1, κ	304008
E2	CD45RA	HI100	Mouse IgG2b, κ	304108
E3	CD45RB	MEM-55	Mouse IgG2b, κ	310204
E4	CD45RO	UCHL1	Mouse IgG2a, κ	304206
E5	CD46	TRA-2–10	Mouse IgG1	352402
E6	CD47	CC2C6	Mouse IgG1, κ	323108
E7	CD48	BJ40	Mouse IgG1, κ	336708
E8	CD49a	TS2/7	Mouse IgG1, κ	328304
E9	CD49c	ASC-1	Mouse IgG1, κ	343804
E10	CD49d	9F10	Mouse IgG1, κ	304304
E11	CD49e	NKI-SAM-1	Mouse IgG2b, κ	328010
E12	CD49f	GoH3	Rat IgG2a, κ	313612
F1	CD50 (ICAM-3)	CBR-IC3/1	Mouse IgG1, κ	330005
F2	CD51	NKI-M9	Mouse IgG2a, κ	327910
F3	CD51/61	23C6	Mouse IgG1, κ	304406
F4	CD52	HI186	Mouse IgG2b, κ	316006
F5	CD53	HI29	Mouse IgG1, κ	325406
F6	CD54	HA58	Mouse IgG1, κ	353106
F7	CD55	JS11	Mouse IgG1, κ	311308
F8	CD56 (NCAM)	HCD56	Mouse IgG1, κ	318306
F9	CD57	HCD57	Mouse IgM, κ	322312
F10	CD58	TS2/9	Mouse IgG1, κ	330905
F11	CD59	p282 (H19)	Mouse IgG2a, κ	304708
F12	CD61	VI-PL2	Mouse IgG1, κ	336406
G1	CD62E	HAE-1f	Mouse IgG1, κ	336008
G2	CD62L	DREG-56	Mouse IgG1, κ	304806
G3	CD62P (P-Selectin)	AK4	Mouse IgG1, κ	304906
G4	CD63	H5C6	Mouse IgG1, κ	353004
G5	CD64	10.1	Mouse IgG1, κ	305008
G6	CD66a/c/e	ASL-32	Mouse IgG2b, κ	342304
G7	CD66b	G10F5	Mouse IgM, κ	305106
G8	CD69	FN50	Mouse IgG1, κ	310906
G9	CD70	113-16	Mouse IgG1, κ	355104
G10	CD71	CY1G4	Mouse IgG2a, κ	334106
G11	CD73	AD2	Mouse IgG1, κ	344004
G12	CD74	LN2	Mouse IgG1, κ	326808
H1	CD79b	CB3-1	Mouse IgG1, κ	341404
H2	CD80	2D10	Mouse IgG1, κ	305208
H3	CD81	5A6	Mouse IgG1, κ	349506
H4	CD82	ASL-24	Mouse IgG1, κ	342104
H5	CD83	HB15e	Mouse IgG1, κ	305308
H6	CD84	CD84.1.21	Mouse IgG2a, κ	326008
H7	CD85a (ILT5)	MKT5.1	Rat IgG2a, κ	337704
H8	CD85d (ILT4)	42D1	Rat IgG2a, κ	338706
H9	CD85g (ILT7)	17G10.2	Mouse IgG1, κ	326408
H10	CD85h (ILT1)	24	Mouse IgG2b, κ	337904
H11	CD85j (ILT2)	GHI/75	Mouse IgG2b, κ	333708
H12	CD85k (ILT3)	ZM4.1	Mouse IgG1, κ	333008
				
*LEGENDSCREEN PLATE #2*				
A1	Blank			
A2	CD86	IT2.2	Mouse IgG2b, κ	305406
A3	CD87	VIM5	Mouse IgG1, κ	336906
A4	CD88	S5/1	Mouse IgG2a, κ	344304
A5	CD89	A59	Mouse IgG1, κ	354104
A6	CD90 (Thy1)	5E10	Mouse IgG1, κ	328110
A7	CD93	VIMD2	Mouse IgG1, κ	336108
A8	CD94	DX22	Mouse IgG1, κ	305506
A9	CD95	DX2	Mouse IgG1, κ	305608
A10	CD96	NK92.39	Mouse IgG1, κ	338406
A11	CD97	VIM3b	Mouse IgG1, κ	336308
A12	CD99	HCD99	Mouse IgG2a, κ	318008
B1	CD100	A8	Mouse IgG1, κ	328408
B2	CD101 (BB27)	BB27	Mouse IgG1, κ	331006
B3	CD102	CBR-IC2/2	Mouse IgG2a, κ	328506
B4	CD103	Ber-ACT8	Mouse IgG1, κ	350206
B5	CD104	58XB4	Mouse IgG2a, κ	327808
B6	CD105	43A3	Mouse IgG1, κ	323206
B7	CD106	STA	Mouse IgG1, κ	305806
B8	CD107a (LAMP-1)	H4A3	Mouse IgG1, κ	328608
B9	CD108	MEM-150	Mouse IgM, κ	315704
B10	CD109	W7C5	Mouse IgG1, κ	323306
B11	CD111	R1.302	Mouse IgG1, κ	340404
B12	CD112 (Nectin-2)	TX31	Mouse IgG1, κ	337410
C1	CD114	LMM741	Mouse IgG1, κ	346106
C2	CD115	9-4D2-1E4	Rat IgG1, κ	347304
C3	CD116	4H1	Mouse IgG1, κ	305908
C4	CD117 (c-kit)	104D2	Mouse IgG1, κ	313204
C5	CD119 (IFN-g R α chain)	GIR-208	Mouse IgG1, κ	308606
C6	CD122	TU27	Mouse IgG1, κ	339006
C7	CD123	6H6	Mouse IgG1, κ	306006
C8	CD124	G077F6	Mouse IgG2a, κ	355004
C9	CD126 (IL-6Rα)	UV4	Mouse IgG1, κ	352804
C10	CD127 (IL-7Rα)	A019D5	Mouse IgG1, κ	351304
C11	CD129 (IL-9 R)	AH9R7	Mouse IgG2b, κ	310404
C12	CD131	1C1	Mouse IgG1, κ	306104
D1	CD132	TUGh4	Rat IgG2b, κ	338606
D2	CD134	Ber-ACT35 (ACT35)	Mouse IgG1, κ	350004
D3	CD135	BV10A4H2	Mouse IgG1, κ	313306
D4	CD137 (4-1BB)	4B4-1	Mouse IgG1, κ	309804
D5	CD137L (4-1BB Ligand)	5F4	Mouse IgG1, κ	311504
D6	CD138	DL-101	Mouse IgG1, κ	352306
D7	CD140a	16A1	Mouse IgG1, κ	323506
D8	CD140b	18A2	Mouse IgG1, κ	323606
D9	CD141	M80	Mouse IgG1, κ	344104
D10	CD143	5–369	Mouse IgG1, κ	344204
D11	CD144	BV9	Mouse IgG2a, κ	348506
D12	CD146	SHM-57	Mouse IgG2a, κ	342004
E1	CD148	A3	Mouse IgG1, κ	328708
E2	CD150 (SLAM)	A12 (7D4)	Mouse IgG1, κ	306308
E3	CD152	L3D10	Mouse IgG1, κ	349906
E4	CD154	24–31	Mouse IgG1, κ	310806
E5	CD155 (PVR)	SKII.4	Mouse IgG1, κ	337610
E6	CD156c (ADAM10)	SHM14	Mouse IgG1, κ	352704
E7	CD158a/h	HP-MA4	Mouse IgG2b, κ	339506
E8	CD158b (KIR2DL2/L3, NKAT2)	DX27	Mouse IgG2a, κ	312606
E9	CD158d	mAb 33 (33)	Mouse IgG1, κ	347006
E10	CD158e1 (KIR3DL1, NKB1)	DX9	Mouse IgG1, κ	312708
E11	CD158f	UP-R1	Mouse IgG1, κ	341304
E12	CD161	HP-3G10	Mouse IgG1, κ	339904
F1	CD162	KPL-1	Mouse IgG1, κ	328806
F2	CD163	GHI/61	Mouse IgG1, κ	333606
F3	CD164	67D2	Mouse IgG1, κ	324808
F4	CD165	SN2 (N6- D11)	Mouse IgG1, κ	329010
F5	CD166	3A6	Mouse IgG1, κ	343904
F6	CD167a (DDR1)	51D6	Mouse IgG3, κ	334006
F7	CD169	7–239	Mouse IgG1, κ	346004
F8	CD170 (Siglec-5)	1A5	Mouse IgG1, κ	352004
F9	CD172a (SIRPa)	SE5A5	Mouse IgG1, κ	323806
F10	CD172b (SIRPb)	B4B6	Mouse IgG1, κ	323906
F11	CD172g (SIRPg)	LSB2.20	Mouse IgG1, κ	336606
F12	CD178 (Fas-L)	NOK-1	Mouse IgG1, κ	306407
G1	CD179a	HSL96	Mouse IgG1, κ	347404
G2	CD179b	HSL11	Mouse IgG1, κ	349804
G3	CD180 (RP105)	MHR73-11	Mouse IgG1, κ	312906
G4	CD181 (CXCR1)	8F1/CXCR1	Mouse IgG2b, κ	320608
G5	CD182 (CXCR2)	5E8/CXCR2	Mouse IgG1, κ	320706
G6	CD183	G025H7	Mouse IgG1, κ	353706
G7	CD184 (CXCR4)	12G5	Mouse IgG2a, κ	306506
G8	CD193 (CCR3)	5E8	Mouse IgG2b, κ	310706
G9	CD195 (CCR5)	T21/8	Mouse IgG1, κ	321406
G10	CD196	G034E3	Mouse IgG2b, κ	353410
G11	CD197 (CCR7)	G043H7	Mouse IgG2a, κ	353204
G12	CD200 (OX2)	OX-104	Mouse IgG1, κ	329206
H1	CD200 R	OX-108	Mouse IgG1, κ	329306
H2	CD201 (EPCR)	RCR-401	Rat IgG1, κ	351904
H3	CD202b ( Tie2/Tek)	33.1 (Ab33)	Mouse IgG1, κ	334206
H4	CD203c (E-NPP3)	NP4D6	Mouse IgG1, κ	324606
H5	CD205 (DEC- 205)	HD30	Mouse IgG1, κ	342204
H6	CD206 (MMR)	15-2	Mouse IgG1, κ	321106
H7	CD207 (Langerin)	10E2	Mouse IgG1, κ	352204
H8	CD209 (DC- SIGN)	9E9A8	Mouse IgG2a, κ	330106
H9	CD210 (IL- 10 R)	3F9	Rat IgG2a, κ	308804
H10	CD213a2	SHM38	Mouse IgG1, κ	354404
H11	CD215 (IL- 15Rα)	JM7A4	Mouse IgG2b, κ	330208
H12	CD218a (IL-18Rα)	H44	Mouse IgG1, κ	313808
				
*LEGENDSCREEN PLATE #3*				
A1	Blank			
A2	CD220	B6.220	Mouse IgG2b, κ	352604
A3	CD221 (IGF-1R)	1H7/CD221	Mouse IgG1, κ	351806
A4	CD226 (DNAM-1)	11A8	Mouse IgG1, κ	338306
A5	CD229 (Ly-9)	HLy-9.1.25	Mouse IgG1, κ	326108
A6	CD231 (TALLA)	SN1a (M3- 3D9)	Mouse IgG1, κ	329406
A7	CD235ab	HIR2	Mouse IgG2b, κ	306604
A8	CD243	UIC2	Mouse IgG2a, κ	348606
A9	CD244 (2B4)	C1.7	Mouse IgG1, κ	329508
A10	CD245 (p220/240)	DY12	Mouse IgG1, κ	Inquire
A11	CD252 (OX40L)	11C3.1	Mouse IgG1, κ	326308
A12	CD253 (Trail)	RIK-2	Mouse IgG1, κ	308206
B1	CD254	MIH24	Mouse IgG1, κ	347504
B2	CD255 (TWEAK)	CARL-1	Mouse IgG3, κ	308305
B3	CD257 (BAFF, BLYS)	T7–241	Mouse IgG1, κ	318606
B4	CD258 (LIGHT)	T5–39	Mouse IgG2a, κ	318706
B5	CD261 (DR4, TRAIL-R1)	DJR1	Mouse IgG1, κ	307206
B6	CD262 (DR5, TRAIL-R2)	DJR2–4 (7–8)	Mouse IgG1, κ	307406
B7	CD263 (DcR1, TRAIL-R3)	DJR3	Mouse IgG1, κ	307006
B8	CD266 (Fn14, TWEAK Receptor)	ITEM-1	Mouse IgG1, κ	314004
B9	CD267 (TACI)	1A1	Rat IgG2a, κ	311906
B10	CD268 (BAFF-R, BAFFR)	11C1	Mouse IgG1, κ	316906
B11	CD270 (HVEM)	122	Mouse IgG1, κ	318806
B12	CD271	ME20.4	Mouse IgG1, κ	345106
C1	CD273 (B7- DC, PD-L2)	24F.10C12	Mouse IgG2a, κ	329606
C2	CD274 (B7- H1, PD-L1)	29E.2A3	Mouse IgG2b, κ	329706
C3	CD275 (B7- H2, B7-RP1, ICOSL)	9F.8A4	Mouse IgG1, κ	329806
C4	CD276	MIH42	Mouse IgG1, κ	351004
C5	CD277	BT3.1	Mouse IgG1, κ	342704
C6	CD278 (ICOS)	C398.4A	Arm. Hamster IgG	313508
C7	CD279 (PD-1)	EH12.2H7	Mouse IgG1, κ	329906
C8	CD282 (TLR2)	TL2.1	Mouse IgG2a, κ	309708
C9	CD284 (TLR4)	HTA125	Mouse IgG2a, κ	312806
C10	CD286 (TLR6)	TLR6.127	Mouse IgG1, κ	334708
C11	CD290	3C10C5	Mouse IgG1, κ	354604
C12	CD294	BM16	Rat IgG2a, κ	350106
D1	CD298	LNH-94	Mouse IgG1, κ	341704
D2	CD300e (IREM-2)	UP-H2	Mouse IgG1, κ	339704
D3	CD300F	UP-D2	Mouse IgG1, κ	340604
D4	CD301	H037G3	Mouse IgG2a, κ	354704
D5	CD303	201A	Mouse IgG2a, κ	354204
D6	CD304	12C2	Mouse IgG2a, κ	354504
D7	CD307	509f6	Mouse IgG2a, κ	340304
D8	CD307d (FcRL4)	413D12	Mouse IgG2b, κ	340204
D9	CD314 (NKG2D)	1D11	Mouse IgG1, κ	320806
D10	CD317	RS38E	Mouse IgG1, κ	348406
D11	CD318 (CDCP1)	CUB1	Mouse IgG2b, κ	324006
D12	CD319 (CRACC)	162.1	Mouse IgG2b, κ	331806
E1	CD324 (E- Cadherin)	67A4	Mouse IgG1, κ	324106
E2	CD325	8C11	Mouse IgG1, κ	350805
E3	CD326 (Ep- CAM)	9C4	Mouse IgG2b, κ	324206
E4	CD328 (Siglec-7)	6–434	Mouse IgG1, κ	339204
E5	CD334 (FGFR4)	4FR6D3	Mouse IgG1, κ	324306
E6	CD335 (NKp46)	9E2	Mouse IgG1, κ	331908
E7	CD336 (NKp44)	P44-8	Mouse IgG1, κ	325108
E8	CD337 (NKp30)	P30-15	Mouse IgG1, κ	325208
E9	CD338 (ABCG2)	5D3	Mouse IgG2b, κ	332008
E10	CD340 (erbB2/ HER-2)	24D2	Mouse IgG1, κ	324406
E11	CD344 (Frizzled-4)	CH3A4A7	Mouse IgG1, κ	326606
E12	CD351	TX61	Mouse IgG1, κ	137306
F1	CD352 (NTB-A)	NT-7	Mouse IgG1, κ	317208
F2	CD354 (TREM-1)	TREM-26	Mouse IgG1, κ	314906
F3	CD355 (CRTAM)	Cr24.1	Mouse IgG2a, κ	339106
F4	CD357 (GITR)	621	Mouse IgG1, κ	311604
F5	CD360 (IL- 21R)	2G1-K12	Mouse IgG1, κ	347806
F6	β2- micro- globulin	2M2	Mouse IgG1, κ	316306
F7	BTLA	MIH26	Mouse IgG2a, κ	344506
F8	C3AR	hC3aRZ8	Mouse IgG2b	345804
F9	C5L2	1D9-M12	Mouse IgG2a, κ	342404
F10	CCR10	5/1/6588	Arm. hamster IgG	341504
F11	CLEC12A	50C1	Mouse IgG2a, κ	353604
F12	CLEC9A	8F9	Mouse IgG2a, κ	353804
G1	CX3CR1	2A9-1	Rat IgG2b, κ	341604
G2	CXCR7	8F11-M16	Mouse IgG2b, κ	331104
G3	δ-Opioid Receptor	DOR7D2A4	Mouse IgG2b, κ	327206
G4	DLL1	MHD1–314	Mouse IgG1, κ	346404
G5	DLL4	MHD4–46	Mouse IgG1, κ	346506
G6	DR3 (TRAMP)	JD3	Mouse IgG1, κ	307106
G7	EGFR	AY13	Mouse IgG1, κ	352904
G8	erbB3/HER-3	1B4C3	Mouse IgG2a, κ	324706
G9	FcεRIα	AER-37 (CRA-1)	Mouse IgG2b, κ	334610
G10	FcRL6	2H3	Mouse IgG2b, κ	Inquire
G11	Galectin-9	9M1-3	Mouse IgG1, κ	348906
G12	GARP (LRRC32)	7B11	Mouse IgG2b, κ	352504
H1	HLA-A,B,C	W6/32	Mouse IgG2a, κ	311406
H2	HLA-A2	BB7.2	Mouse IgG2b, κ	343306
H3	HLA-DQ	HLADQ1	Mouse IgG1, κ	318106
H4	HLA-DR	L243	Mouse IgG2a, κ	307606
H5	HLA-E	3D12	Mouse IgG1, κ	342604
H6	HLA-G	87G	Mouse IgG2a, κ	335906
H7	IFN-g R b chain	2HUB-159	Hamster IgG	308504
H8	Ig light chain k	MHK-49	Mouse IgG1, κ	316508
H9	Ig light chain λ	MHL-38	Mouse IgG2a, κ	316608
H10	IgD	IA6-2	Mouse IgG2a, κ	348204
H11	IgM	MHM-88	Mouse IgG1, κ	314508
H12	IL-28RA	MHLICR2a	Mouse IgG2a, κ	337804
				
*LEGENDSCREEN PLATE #4*				
A1	Blank			
A2	Integrin α9β1	Y9A2	Mouse IgG1, κ	351606
A3	integrin β5	AST-3T	Mouse IgG2a, κ	345204
A4	integrin β7	FIB504	Rat IgG2a, κ	321204
A5	Jagged 2	MHJ2–523	Mouse IgG1, κ	346904
A6	LAP	TW4-6H10	Mouse IgG1, κ	349704
A7	Lymphotoxin b Receptor (LT-bR)	31G4D8	Mouse IgG2b, κ	322008
A8	Mac-2 (Ga- lectin-3)	Gal397	Mouse IgG1, κ	126705
A9	MAIR-II	TX45	Mouse IgG1, κ	334804
A10	MICA/MICB	6D4	Mouse IgG2a, κ	320906
A11	MSC (W3D5)	W3D5	Mouse IgG2a, κ	327506
A12	MSC (W5C5)	W5C5	Mouse IgG1, κ	327406
B1	MSC (W7C6)	W7C6	Mouse IgG1, κ	327606
B2	MSC and NPC (W4A5)	W4A5	Mouse IgG1, κ	330806
B3	MSCA-1 (MSC, W8B2)	W8B2	Mouse IgG1, κ	327306
B4	NKp80	5D12	Mouse IgG1, κ	346706
B5	Notch 1	MHN1–519	Mouse IgG1, κ	352106
B6	Notch 2	MHN2–25	Mouse IgG2a, κ	348304
B7	Notch 3	MHN3–21	Mouse IgG1, κ	345406
B8	Notch 4	MHN4-2	Mouse IgG1, κ	349004
B9	NPC (57D2)	57D2	Mouse IgG1, κ	327706
B10	Podoplanin	NC-08	Rat IgG2a, λ	337004
B11	Pre-BCR	HSL2	Mouse IgG1, κ	347904
B12	PSMA	LNI-17	Mouse IgG1, κ	342504
C1	Siglec-10	5G6	Mouse IgG1, κ	347604
C2	Siglec-8	7C9	Mouse IgG1, κ	347104
C3	Siglec-9	K8	Mouse IgG1, κ	351504
C4	SSEA-1	MC-480	Mouse IgM, κ	125606
C5	SSEA-3	MC-631	Rat IgM, κ	330312
C6	SSEA-4	MC-813-70	Mouse IgG3, κ	330406
C7	SSEA-5	8.00E+11	Mouse IgG1, κ	355204
C8	TCR g/d	B1	Mouse IgG1, κ	331210
C9	TCR Vβ13.2	H132	Mouse IgG1, κ	333108
C10	TCR Vβ23	αHUT7	Mouse IgG1, κ	349406
C11	TCR Vβ8	JR2 (JR.2)	Mouse IgG2b, κ	348104
C12	TCR Vβ9	MKB1	Mouse IgG2b, κ	349204
D1	TCR Vδ2	B6	Mouse IgG1, κ	331408
D2	TCR Vg9	B3	Mouse IgG1, κ	331308
D3	TCR Vα24- Jα18	6B11	Mouse IgG1, κ	342904
D4	TCR Vα7.2	3C10	Mouse IgG1, κ	351706
D5	TCR α/β	IP26	Mouse IgG1, κ	306708
D6	Tim-1	1D12	Mouse IgG1, κ	353904
D7	Tim-3	F38-2E2	Mouse IgG1, κ	345006
D8	Tim-4	9F4	Mouse IgG1, κ	354004
D9	TLT-2	MIH61	Mouse IgG1, κ	351104
D10	TRA-1-60-R	TRA-1-60-R	Mouse IgM, κ	330610
D11	TRA-1–81	TRA-1–81	Mouse IgM, κ	330708
D12	TSLPR (TSLP-R)	1B4	Mouse IgG1, κ	322806
E1	Ms IgG1, κ ITCL	MOPC-21	Mouse IgG1, κ	400112
E2	Ms IgG2a, κ ITCL	MOPC-173	Mouse IgG2a, κ	400212
E3	Ms IgG2b, κ ITCL	MPC-11	Mouse IgG2b, κ	400314
E4	Ms IgG3, κ ITCL	MG3–35	Mouse IgG3, κ	401320
E5	Ms IgM, κ ITCL	MM-30	Mouse IgM, κ	401609
E6	Rat IgG1, κ ITCL	RTK2071	Rat IgG1, κ	400408
E7	Rat IgG2a, κ ITCL	RTK2758	Rat IgG2a, κ	400508
E8	Rat IgG2b, κ ITCL	RTK4530	Rat IgG2b, κ	400636
E9	Rat IgM, κ ITCL	RTK2118	Rat IgM, κ	400808
E10	AH IgG, ITCL	HTK888	Arm. Hamster IgG	400907
E11	Blank			
E12	Blank			
F1	Blank			
F2	Blank			
F3	Blank			
F4	Blank			
F5	Blank			
F6	Blank			
F7	Blank			
F8	Blank			
F9	Blank			
F10	Blank			
F11	Blank			
F12	Blank			
G1	Blank			
G2	Blank			
G3	Blank			
G4	Blank			
G5	Blank			
G6	Blank			
G7	Blank			
G8	Blank			
G9	Blank			
G10	Blank			
G11	Blank			
G12	Blank			
H1	Blank			
H2	Blank			
H3	Blank			
H4	Blank			
H5	Blank			
H6	Blank			
H7	Blank			
H8	Blank			
H9	Blank			
				
*Plate 4*				
Well ID	Specificity	Clone	Isotype	BioLegend Cat. No.
H10	Blank			
H11	Blank			
H12	Blank			
